# Genomic Plasticity Enables Phenotypic Variation of *Pseudomonas syringae* pv. *tomato* DC3000

**DOI:** 10.1371/journal.pone.0086628

**Published:** 2014-02-06

**Authors:** Zhongmeng Bao, Paul V. Stodghill, Christopher R. Myers, Hanh Lam, Hai-Lei Wei, Suma Chakravarthy, Brian H. Kvitko, Alan Collmer, Samuel W. Cartinhour, Peter Schweitzer, Bryan Swingle

**Affiliations:** 1 Department of Plant Pathology and Plant-Microbe Biology, Cornell University, Ithaca, New York, United States of America; 2 United States Department of Agriculture-Agricultural Research Service, Ithaca, New York, United States of America; 3 Department of Physics, Laboratory of Atomic and Solid State Physics, Cornell University, Ithaca, New York, United States of America; 4 MSU-DOE Plant Research Laboratory, Michigan State University, East Lansing, Michigan, United States of America; 5 Biotechnology Resource Center, Cornell University, Ithaca, New York, United States of America; University of the West of England, United Kingdom

## Abstract

Whole genome sequencing revealed the presence of a genomic anomaly in the region of 4.7 to 4.9 Mb of the *Pseudomonas syringae* pv. *tomato* (*Pst*) DC3000 genome. The average read depth coverage of *Pst* DC3000 whole genome sequencing results suggested that a 165 kb segment of the chromosome had doubled in copy number. Further analysis confirmed the 165 kb duplication and that the two copies were arranged as a direct tandem repeat. Examination of the corresponding locus in *Pst* NCPPB1106, the parent strain of *Pst* DC3000, suggested that the 165 kb duplication most likely formed after the two strains diverged via transposition of an ISPsy5 insertion sequence (IS) followed by unequal crossing over between ISPsy5 elements at each end of the duplicated region. Deletion of one copy of the 165 kb region demonstrated that the duplication facilitated enhanced growth in some culture conditions, but did not affect pathogenic growth in host tomato plants. These types of chromosomal structures are predicted to be unstable and we have observed resolution of the 165 kb duplication to single copy and its subsequent re-duplication. These data demonstrate the role of IS elements in recombination events that facilitate genomic reorganization in *P. syringae*.

## Introduction

Genetic diversity within populations underpins the process of natural selection, enabling individuals with particular genotypes to outgrow competitors, survive stresses and invade or specialize for growth in different environments. Unlike eukaryotes that have the benefit of meiotic recombination, genetic variation in prokaryotes is not mechanistically tied with reproduction, but instead, is achieved through horizontal transfer [1], [2], [3], [4], mutation [5], [6] and recombination [7], [8], [9], [10]. This strategy works for prokaryotes because their unicellular nature allows genetic diversity to arise within a population and for natural selection to act upon each cell, permitting evolution to advance by directly selecting the fittest individuals from the population [11].

Plant pathogenic bacteria have adapted to the specific challenges associated with invading and colonizing plant tissues. In most plant pathogenic bacteria the pathogenic process involves Type Three secretion system-dependent injection of virulence effectors into host cells, which interfere with the plant's ability to detect infection and disrupt the plant's immune defenses [12]. Colonization and disease depend on the bacteria injecting the correct combination of effectors that prevent or suppress the plant's defenses without provoking an unchecked immune response. The relationship between bacterial virulence mechanisms and plant immune systems can be portrayed as an evolutionary arms race, in which the plant immune and microbe virulence systems continuously adapt to each other [13].

*Pseudomonas syringae* is a complex of Gram-negative **γ**-proteobacteria that exist in diverse environments and cause disease in many plants [14]. *P. syringae* is divided into more than 50 distinct pathovars [15] and further subdivided into races based on the range of host species and cultivars that individual strains can infect. Host specificity is partially determined by the combination of effectors produced, and can change by altering the effector gene repertoire [16], [17]. Furthermore, it now appears that wild (non-domesticated) populations of *P. syringae* may exist as generalists and adapt away from this lifestyle to specialize as aggressive host-specific pathogens in agricultural monoculture systems [18]. In the process of adapting to new hosts, the bacteria may be exposed to antimicrobial plant defenses that select for mutants with compatible effector repertoires. The fastest mechanisms to permit adaptation involve recombination [19] and genomic rearrangement [20], in some cases mediated by transposition or other mobile DNA elements [16], [21], [22]. These sorts of genomic events are much more frequent than beneficial point mutations [23], [24] and allow the organism to make evolutionary leaps, changing phenotypes dramatically in a single event [20], [25].

Several *P. syringae* strains have emerged as tractable models used extensively to investigate the molecular mechanisms of plant-microbe interactions [26], [27], [28]. One widely used model strain is *P. syringae* pv. *tomato* (*Pst*) DC3000, the first member of the *P. syringae* group for which a complete, closed genome sequence was reported [29]. Here we identify and characterize a dynamic, 165 kb genomic duplication in *Pst* DC3000 that can influence growth of the bacteria in different conditions. We used whole genome sequence-assisted copy number analysis to identify the 165 kb duplicated region, and determined the structural organization and genomic location using PCR and recombineering. Our results show that this duplication is present in some laboratory isolates including the *Pst* DC3000 isolate used to produce the reference sequence [29] and in the *Pst* DC3000 strain commercially available from the American Type Culture Collection (ATCC). However, the 165 kb duplication is not present in *Pst* NCPPB1106, the strain from which *Pst* DC3000 is derived, indicating that the duplication arose after the two strains diverged in the mid-1980's [26], [30]. The 165 kb duplication has the structure of a typical tandem repeat [31] in which each copy is separated from the other and flanked on each end by an ISPsy5 insertion sequence (IS). These observations provide evidence that homologous recombination between identical IS elements can facilitate genomic flexibility in *Pst* DC3000 and suggest a mechanism by which bacteria can adapt to diverse and changing environments.

## Materials and Methods

### Bacterial strains and growth conditions

*P. syringae* strains (Table 1) were grown at 28°C in Kings B (KB) medium [32] or on KB medium solidified with 1.5% (wt/vol) agar. Rifampicin, gentamycin, kanamycin, and tetracycline were used at 50 µg/ml, 10 µg/ml, 50 µg/ml and 10 µg/ml respectively. *Escherichia coli* DH5**α** was used as the host for subcloning and other plasmid manipulations. *E. coli* was grown at 37°C in LB medium or LB medium solidified with 1.5% (wt/vol) agar.

**Table 1:** *P. syringae* pv. *tomato* strains used in this work.

Accession Number^a^	*Dup165* b	Strain	Relevant Characteristics	Reference
SRR1039775	+	*Pst* DC3000(H0457)	*Pst* DC3000 storage no. H0457 in A. Collmer's collection/Rif^R^ strain	[29]
SRR1039776	*+*	*Pst* DC3000(ATCC)	*Pst* DC3000 genomic DNA obtained from ATCC/Rif^R^ strain	ATCC BAA-87
SRR1039777	*+*	*Pst* DC3000(PS1)	*Pst* DC3000 storage no. PS1 in B. Swingle's collection/cultured from *Pst* DC3000(H0457)/Rif^R^ strain	This work
SRR1039778	*+/−*	*Pst* DC3000(JB)	*Pst* DC3000 isolate cultured from *Pst* DC3000(PS1)/Rif^R^ strain	This work
SRR1039717	*+*	*Pst* DC3000(BB)	*Pst* DC3000 isolate cultured from *Pst* DC3000(PS1)/Rif^R^ strain	This work
SRR1039779	*+*	*Pst* DC3000(ZB)	*Pst* DC3000 isolate cultured from *Pst* DC3000(PS1)/Rif^R^ strain	This work
SRR1039780	*+*	*Pst* DC3000(LS)	*Pst* DC3000 isolate cultured from *Pst* DC3000(H0457)/Rif^R^ strain	This work
SRR1039781	*−*	*Pst* DC3000(D36E)	*Pst* DC3000(H0457) with 36 effector genes deleted	This work
SRR1039782	*−*	*Pst* NCPPB1106	Rif^S^ progenitor of *Pst* DC3000; provided by Diane Cuppels	[26], [30]
SRR1039794	*−*	*Pst* DC3000(JF)	*Pst* DC3000 isolate obtained from Jacqueline Fletcher, Oklahoma State University/Rif^R^ strain	This work
NA	*−*	*Pst* DC3000(*dup165* ^−^)	*Pst* DC3000(H0457) with one copy of the 165 kb region deleted/Rif^R^ Kan^R^	This work
NA	*+*	*Pst* DC3000(**Δ**ISPsy5-34)	*Pst* DC3000(H0457) with the central ISPsy5 of the 165 kb tandem duplication deleted/Rif^R^ Kan^R^	This work
NA	*−*	*Pst* DC3000(D28E)	*Pst* DC3000(H0457) with 28 effector genes deleted	[33]
NA	*+*	*Pst* DC3000(D28E **Δ** *iaaL*)	*Pst* DC3000(D28E) with *iaaL* gene deleted	This work

a Sequencing data for isolates analyzed by whole genome sequencing are available at the NCBI Sequence Read Archive.

NA indicates isolate genomes not sequenced.

b *Dup165* indicates presence (+) or absence (−) of the duplication or (+/−) that the evidence of the duplication varied.

*Pst* DC3000(D36E) and *Pst* DC3000(D28E) are referred to as DC3000D36E and DC3000D28E in other work [33].

The *Pst* DC3000(D28E**Δ** *iaaL*) strain was constructed by deletion of the *iaaL* gene from *Pst* DC3000(D28E) [33] using marker exchange mutagenesis [34], [35]. The locus tag for the *iaaL* gene is PSPTO_0371. The deletion construct was generated by cloning 1.2 kb left and 1.3 kb right flanking regions of PSPTO_0371 into pK18mobSacB as described previously [34]. The two flanks were amplified from *Pst* DC3000 genomic DNA using primer pairs HL117/HL118 and HL119/HL120 (see Table S1 for all primer sequences), and then joined by SOEing PCR [36]. The resulting 2.5 kb product was digested with XbaI and ligated with XbaI digested pK18mobsacB. The deletion construct was introduced into *Pst* DC3000(D28E) by electroporation and merodiploid intermediates were selected for growth on medium containing kanamycin. Recombinants that had eliminated pK18mobsacB plasmid sequences were identified by sucrose counter-selection. Sucrose-resistant, Km-sensitive colonies were screened by PCR using primer pair HL121/HL122. The mutation was confirmed by Sanger sequencing with primers HL121, HL122, HL123, HL124, HL161 and HL162.

For complementation, the *iaaL* gene was cloned into the *P. syringae* expression vector pBS46 for constitutive overexpression driven by the *PnptII* promoter. The *iaaL* gene from *Pst* DC3000 was cloned into pBS46 using Invitrogen Gateway cloning system as described in [37]. The *iaaL* cloned in pENTR/SD/D was sequenced using primers shown in Table S1.

### PCR

The structural organization of genomic loci was probed using PCR and Sanger sequencing. PCRs were carried out using Takara ExTaq premix. PCR products were resolved on 1% agarose gels, Roche DNA Molecular Weight Marker X was used to estimate length of PCR products. All Sanger sequencing was performed by the Cornell University Biotechnology Resource Center (BRC), Ithaca, NY.

### Growth curves

Growth of *P. syringae* strains was analyzed in a Bioscreen automated growth curve analysis system. For these experiments, *P. syringae* strains were grown overnight at 30°C with vigorous shaking in liquid KB medium with appropriate antibiotics. The overnight cultures were diluted to OD_600_ of 0.2 and 200 µl the diluted overnights were cultured in the Bioscreen for 48 hours at 28°C with continuous shaking and OD_600_ measured every 15 minutes. Results shown are the average of three biological replicates.

### Analysis of growth curves

The growth curve data for *Pst* DC3000 and *Pst* DC3000(*dup165* ^−^) grown in KB were further analyzed to estimate the growth rates of each strain at early times during log phase. The growth curve data do not conform to simple mathematical models of bacterial growth [38], as there is no regime where bacterial growth is strictly exponential in time, as would be expected in log phase (see Figure S1). Nevertheless, both strains grew approximately exponentially at early times with instantaneous growth rates that remained in roughly constant proportion over the first 6 hours of growth. To quantitatively estimate a growth rate difference at early times, we performed the following analysis. First, taking the OD_600_ readings as proxy for the population size N in each sample, we normalized each growth curve by its initial population size N_0_ at time t = 0, and log-transformed the normalized data to obtain curves of log_2_(N/N_0_) vs t. The local slope of these log-transformed growth curves reflects the instantaneous growth rate at each point in time; we approximated the local slope numerically by computing a finite-difference approximation to the derivative of each curve at each time point (main part of Figure S1). Since computing finite-difference derivatives and ratios of data sets increases noise, we also repeated this same procedure with smoothed versions of the log-transformed growth data (boxcar window of width 10, red curve in inset), yielding an average growth rate ratio of 1.44±0.02. We thus estimate a 44% increase in growth rate for the wild-type *Pst* DC3000 strain at early times when both strains were growing approximately exponentially.

### Sequencing and analysis

Whole genome sequencing was provided by the Cornell University BRC, Ithaca, NY. Library preparation and sequencing were performed according to the protocols provided by Illumina. The *Pst* DC3000(H0457), *Pst* DC3000(ATCC), *Pst* DC3000(ZB), *Pst* DC3000(LS), *Pst* DC3000(JB), *Pst* DC3000(BB), *Pst* DC3000(PS1) genomic DNA sequencing libraries were prepared using Illumina Genome Analyzer v4 sequencing kits, Genome Analyzer v4 cluster kits and sequenced on an Illumina Genome Analyzer GAIIx, using single-end 86 bp sequencing. *Pst* DC3000(D36E) and *Pst* DC3000(JF) genomic DNA sequencing libraries were prepared using Nextera XT DNA Sample Preparation Kit (24 samples, FC-131-1024) with Nextera XT Index Kit (FC-131-1001). The *Pst* NCPPB1106 genomic DNA sequencing libraries were prepared using Illumina TruSeq sample prep kit v2 and indexed with TruSeq indexed adaptor #1 (ATCACG). The *Pst* NCPPB1106, *Pst* DC3000(D36E) and *Pst* DC3000(JF) were sequenced using single-end 100 bp, using Illumina v3 cluster kits and SBS reagent kits on an Illumina HiSeq 2000, with HiSeq control software (HCS) version 1.5.15.1 and Real Time Analysis (RTA) software version 1.13.48.

### Copy number analysis

Sinister profiles were constructed from raw high-throughput sequencing reads using the method described in [39], with the modification that the alignment was computed using Bowtie2 version 2.1.0 [40] with the following non-default parameters, –local -L 31 –ma 1 –mp 3 –np 0 –rdg 2,3 –rfg 2,3 -k 2000 -i S,1,0.25 –score-min L,0,0.9. As described in [39], reads that could be aligned to multiple locations were discarded. We then computed a relative sequencibility score for each genomic position. We defined the relative sequencibility score for a base as the sequencibility score (N = 36) [39] for that base as a percentage of the maximum sequencibility score (e.g., if the sequencibility score is 30 out of 36, then the relative sequencibility score is 83%).

The positions in the genome were divided into non-overlapping windows of size 1000. All windows in which less than 80% of the bases had a relative sequencibility score of at least 90% were removed from further consideration.

For each of the remaining windows, we computed the average number of reads starting within that window (i.e., values in the sinister plot) for those bases with a relative sequencibility score of at least 90%. We used the log_2_ of the window average in the remainder of the analysis.

We observed that the read count varied across the genome as a result of replication. The closer to the origin, the larger the read count; the closer to the terminus, the lower. In order to account for this variation, we used the model described in [41], namely, that the read counts decrease exponentially as one moved farther from the origin and closer to the terminus. Thus in log-space the expected number of reads across the circular genome can be modeled by a pair of linear functions connecting the expected counts at the original with the expected counts at the terminus. We accounted for the observed variation across the genome in our analysis, as follows.

Using the method described above, we computed log_2_ averages for windows of lengths 1000 or 10,000 centered at the origin and at the terminus. We defined the expected counts across the circular genome as the two lines connecting these two values at these two locations. For each window, we then subtracted the expected read count average from the observed read count average.

In order to generate a list of regions of putative variable copy-number, we computed the mean and standard deviation for the corrected window averages. We declared any window average that was three standard deviations away from the global mean of correct averages to be an “outlier”, and an entry for that window was made in the output list (Table S2).

### Recombineering

Recombineering was performed as described in [42]. Recombineering substrates were produced by PCR and then 500 ng of DNA was used to transform the indicated *P. syringae* strains containing pUCP24/recTE by electroporation. The sequences of all oligos used are shown in Table S1. Oligos were purchased from Integrated DNA Technologies (IDT), Inc., Coralville, IA. PCR products were generated using ExTaq (Takara Bio, Inc, Japan) using the *neo* gene on pK18mobsacB [43] as the template. Fragment size of PCR generated substrates were confirmed by agarose gel electrophoresis and products were purified and concentrated by ethanol precipitation.

### Plant virulence assays

*Solanum lycopersicom* ‘Moneymaker’ plants were germinated and grown in a greenhouse with approximate 16/8 light/dark cycles. Four to five week old plants were inoculated with 3×10^4^ cfu/ml bacterial suspension using blunt syringe infiltration. Bacteria were recovered from plants by taking samples of the leaf tissue at the site of infection using a #2 disk punch (3 disks, total area 0.589 cm^2^) at 2 days post infection (dpi) and 5 dpi. Leaf disks were homogenized by mechanical disruption in 300 µl of 10 mM MgCl. Serial dilutions of the tissue homogenate were plated on the LM agar supplemented with rifampicin and the number of colony forming units per cm^2^ was calculated.

## Results

### Identification of a cryptic duplication in the genome of *Pst* DC3000

ChIP-seq experiments conducted to identify several sigma factor regulons [44], [45] serendipitously revealed evidence of an anomaly in the *Pst* DC3000 genome. Control samples used to demonstrate specific enrichment in these experiments suggested that a large segment of the *Pst* DC3000 genome had a higher than average copy number (Fig. S2). This motivated us to use Illumina methods to re-sequence *Pst* DC3000 used in our laboratories and align the results to the published *Pst* DC3000 reference sequence [29]. The *Pst* DC3000 isolate sequenced in this experiment was prepared from the deep-frozen stock (storage no. H0457) that was cultured and sent by A. Collmer in June of 2000 to The Institute for Genomic Research (TIGR) where it was used to prepare genomic DNA for sequencing *Pst* DC3000 which was reported in Buell et al., 2003 [29].

**Figure 1:**
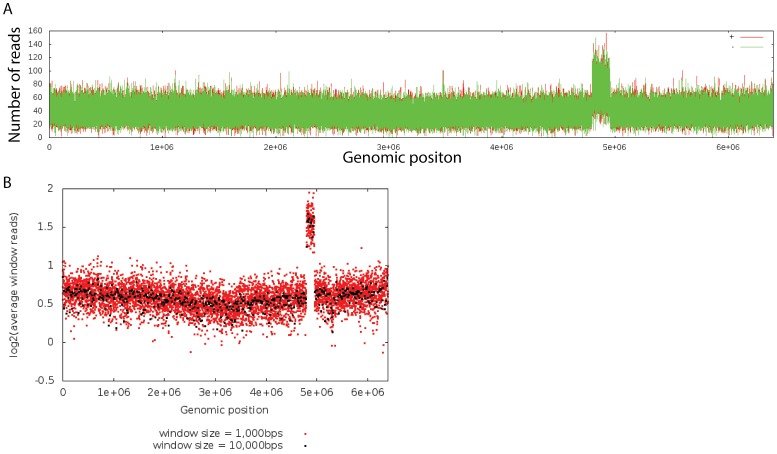
Whole genome sequencing shows a 165*Pst* DC3000. The *Pst* DC3000(H0457) isolate used to generate the reference sequence described by Buell et al., 2003 was re-sequenced using Illumina short read methods. A. Alignment of the observed reads to the reference sequence indicated higher than expected read coverage between genomic coordinates 4790733..4955867. This profile shows the absolute read depth coverage for the *Pst* DC3000 chromosome. Note that the origin of replication (*ori*) is on the left and right ends, and the terminus (*ter*) is in the center, close to position 3×10^6^. Red and green lines show the number of reads matching the positive or negative strand of the chromosome, respectively. B. Copy number analysis indicated that read coverage was increased by two-fold above the expected average. For a complete list of all 1000 bp regions with altered read coverage, see Table S2. The *ori-ter* sequence content gradient is also visible, with more sequence coverage at regions closer to the *ori* as would be expected in growing cells with multiple rounds of replication proceeding concurrently. The region with anomalous coverage is oriented so that one end is closer to the *ter*. We refer to the *ter*-proximal end of the duplicated sequence as the left end and the *ter*-distal end as the right end.

The Illumina sequencing methods used here differ from Sanger sequencing in that the number of reads obtained are proportional to the relative amounts of input DNA. The results of *Pst* DC3000(H0457) re-sequencing were similar to what was observed in the ChIP-seq controls (above) and again showed a region in the vicinity of 4.7 to 4.9 Mbp of the *Pst* DC3000 genome sequence with increased read coverage (Fig. 1A). Specifically, the sequence data indicated that a contiguous region of 165,135 bp located at 4790733..4955867 in the reference sequence [29] had increased read coverage.

Illumina sequencing results for the *Pst* DC3000(H0457) genome were analyzed to determine the degree to which the copy number had been altered in the 4.7 to 4.9 Mbp region. The fold coverage was determined for all 1,000 bp genome sequence blocks relative to an expected local average. The expected local average was computed across the genome to adjust for any differences in read depth due to the origin of replication-to-terminus (*ori-ter*) sequence content gradient (Fig. 1B). The average relative read depth for the entire genome was very close to the expected one-fold read coverage (mean = 1.01 stddev = 0.18). Regions with anomalous coverage were identified by scanning successive 1,000 bp blocks starting at the genomic origin in order to identify windows with relative read depth coverage that differed by more than three standard deviation from the expected local average (Fig. 1B and Table S2). The analysis showed that the portion of the genome between 4790733..4955867 had a read coverage very close to two-fold (mean = 1.94 stdev = 0.20) relative to the expected local average, consistent with duplication of the 165 kb region. Based on these results we refer to this region as the 165 kb duplication throughout the rest of the paper. We note that the data also suggest other genomic regions with less than the expected local average sequence coverage (0.58–0.62) relative fold read coverage, Table S2). We did not examine these regions further, but they may represent deletions within a portion of the sequenced cells or the results may be related to the non-uniform progress of DNA replication.

### The 165 kb duplicated sequences are organized as a tandem duplication

**Figure 2:**
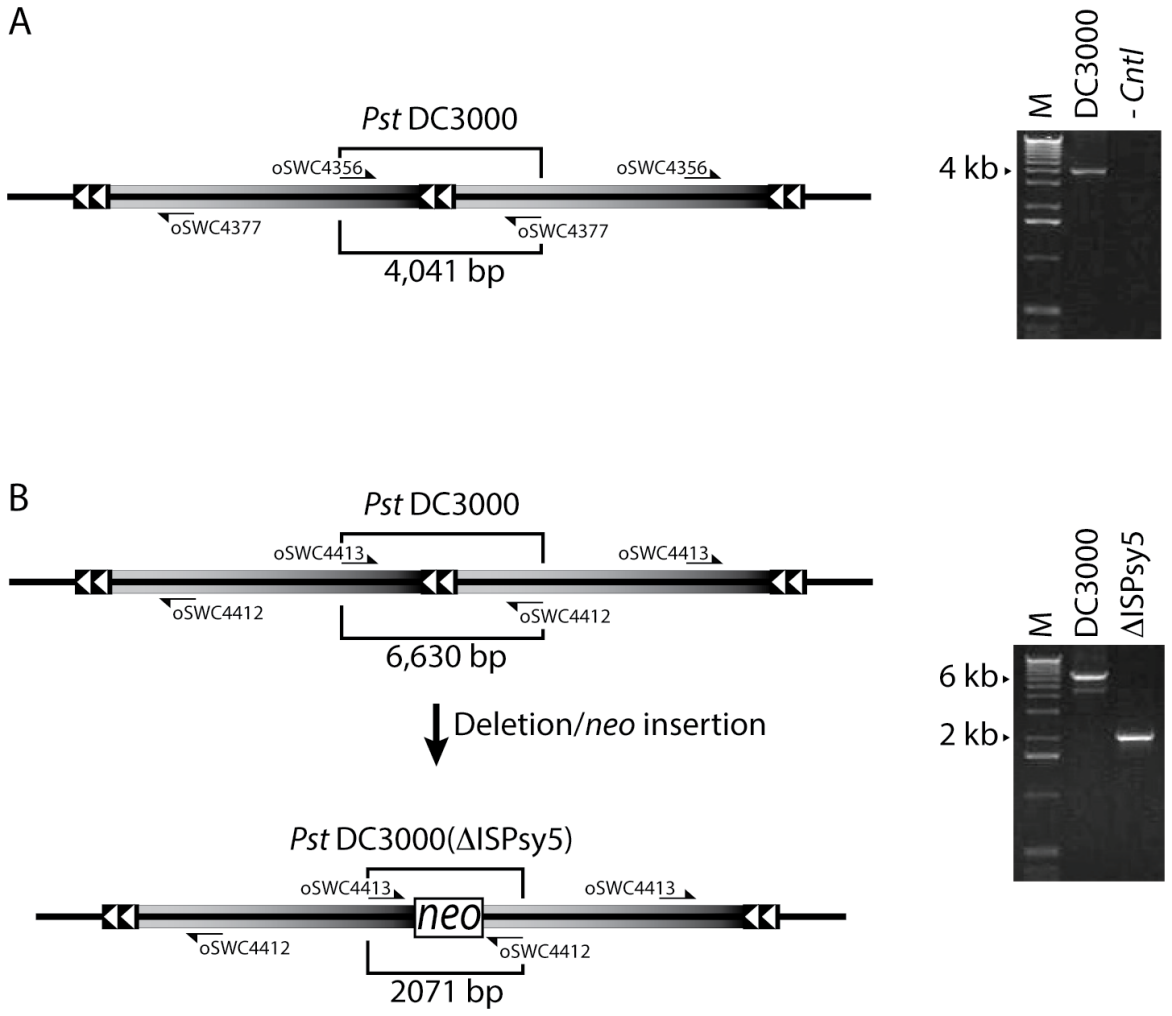
The 165 kb region is a tandem duplication. Each copy of the 165-to-black gradient. The duplicated sequences are flanked by ISPsy5 elements, which are depicted by two white triangles. A. PCR with oSWC4356 and oSWC4377 generate a 4041 bp product when using *Pst* DC3000(H0457) genomic DNA as template. No product is generated using the same primers when genomic DNA from a *Pst* DC3000 isolate lacking the duplication (see below) was used as template (-Cntl). B. The central ISPsy5 element was deleted and replaced with the kanamycin resistance encoding *neo* gene using recombineering. PCR with primers flanking this locus (oSWC4412 and oSWC4413) generate a 6630 bp product when the ISPsy5 element is present and a 2071 bp product in cells where the *neo* gene has replaced the central ISPsy5 element. PCR products were resolved on 1% agarose gel and migration in the predicted size range. M, indicates molecular weight marker.

The 165 kb duplicated sequence is flanked by 2059 bp ISPsy5 insertion sequence (IS) elements oriented as direct repeats relative to each other. The length of these repetitive elements makes it impossible to map the location and orientation of the second copy using only Illumina short read sequence data. Genomic duplications commonly form by unequal crossing over between two homologous IS elements at different locations [31] and are predicted to have a structure similar to that shown in Fig. 2A, with each duplicated segment bounded by repetitive DNA elements. PCR was used to test whether the 165 kb duplication in *Pst* DC3000 had this structure. A product of the predicted size was obtained using primers that anneal at each end of the 165 kb duplicated sequence, consistent with the head-to-tail configuration of a tandem duplication (Fig. 2A).

The arrangement of the 165 kb duplication was also probed using recombineering (Fig. 2B). We reasoned that if the 165 kb duplication had the structure of a tandem duplication, then it should be possible to delete the central ISPsy5 using a recombineering substrate with homology to the 3′ and 5′ ends of each 165 kb copy. *Pst* DC3000 cells containing a *recTE_Psy_* expression plasmid were transformed with a 1305 bp PCR product containing the kanamycin resistance encoding *neo* gene and 80 and 85 bp of homology to regions flanking the central ISPsy5 element. PCR screening of several kanamycin resistant clones indicated that they (12/12) contained the specified *neo* insertion/deletion and two of these clones were further confirmed by sequencing the PCR product. The results show that the central junction was deleted and replaced with the 1.3 kb *neo* gene cassette in these clones exactly as predicted (representative result shown in Fig. 2B). These results confirm a tandem duplication structure, however, it does not address the possibility that the 165 kb region is present as a higher order repeat (i.e., triplication, etc.). In this scenario the deletion of a single junction would leave the other internal ISPsy5 junctions intact. To test this, we examined the kanamycin resistant clones to determine if any retained an internal junction. However, none of 12 recombinants tested generated this type of PCR product. Together, with the copy number analysis (above) these results support the conclusion that the 165 kb region is a tandem duplication and not a higher order amplification.

**Figure 3:**
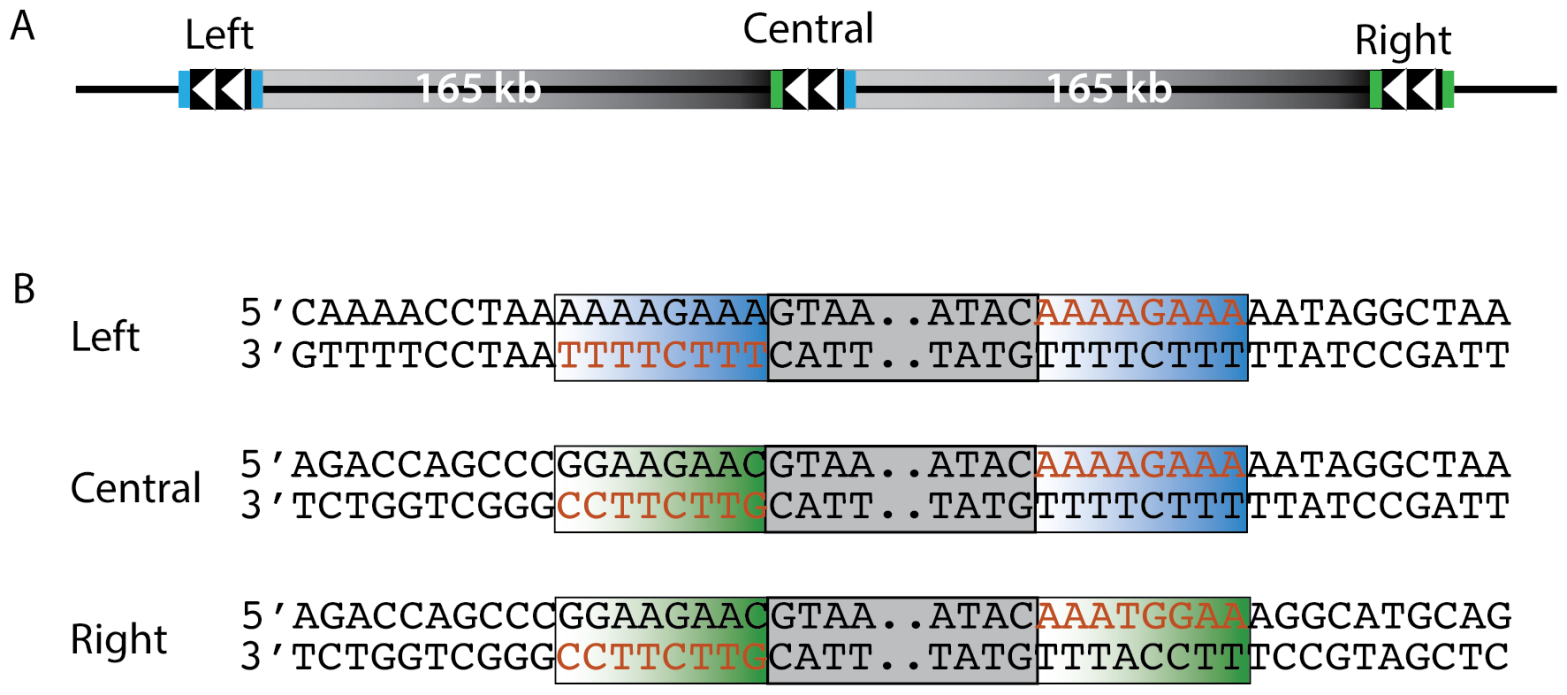
ISPsy5 target site duplications. A. Target site duplications associated with each ISPsy5 element in the 165(blue and green shading) for each ISPsy5 element (gray box, only 4 terminal base pairs are shown) in the 165 kb duplication were determined by Sanger sequencing PCR products that cover each loci.

ISPsy5 is a member of the IS66 family, which generally produces 8 bp target site duplications [46]. The sequences flanking the ISPsy5 elements were analyzed to determine the transposon-genome junctions and the direct repeats produced by the two annotated ISPsy5 insertions flanking the 165 kb duplication. Alignment of the sequences at the ends of the transposon suggests that the ISPsy5 elements have 22 bp imperfect terminal inverted repeats (Fig. S3) and produce 8 bp direct repeats upon insertion (Fig. 3). Production of an 8 bp target site duplication was verified by comparison of the left ISPsy5 locus of *Pst* DC3000 to that of its parent, *Pst* NCPPB1106, which does not have an ISPsy5 at this location or the duplication (see below).

When tandem duplications form by unequal crossing over between IS elements located at different sites on sister chromosomes they are predicted to generate a sequence configuration in which the target site duplication flanking the central element are derived from the two outer IS elements [31] (Fig. 3A). Sequence comparison of the 8 bp target site duplications flanking each ISPsy5 element associated with this tandem duplication confirmed that the central IS element is flanked by direct repeat sequences from the two outer elements, consistent with the duplication having formed by unequal crossing over (Fig. 3B).

### Allelic variation at the 165 kb locus in sequenced *Pst* isolates and derivatives

**Figure 4:**
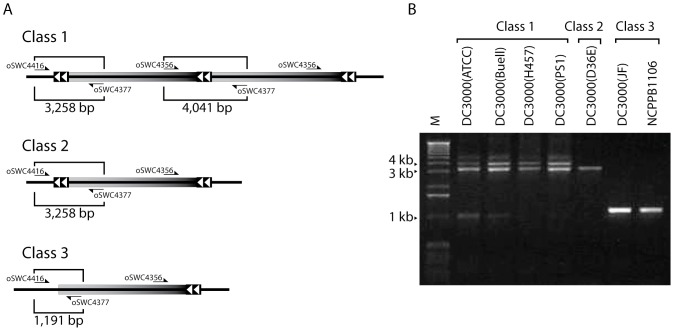
Multiplex PCR demonstrates allelic variation at the 165 kb duplication locus. Three primers (oSWC4416, oSWC4356 and oSWC4377) were used in PCRs with different *Pst* isolates as template. A. Multiplex reactions can distinguish three classes of genetic architecture at the 165 kb duplication locus. Class 1 has the full 165 kb tandem duplication; Class 2 does not have the duplication, but has the pair of ISPsy5 elements framing the 165 kb region; Class 3 does not have the duplication or the ISPsy5 on the left flank of the 165 kb region. B. PCRs resolved on 1% agarose gels show products representing the three varieties of alleles. The genomic DNA used as template in PCRs is shown above each lane. DC3000(Buell) indicates PCR using *Pst* DC3000 genomic DNA that was prepared for the genome sequencing reported in Buell et al., 2003 [29]. All other strains are listed in Table 1. M, molecular weight marker. PCR using oSWC4416 and oSWC4377 occasionally generated minor 1 kb product, as seen in *Pst* DC3000(ATCC) and *Pst* DC3000(Buell). The band was extracted from the gel and sequenced. BLAST analysis indicated that the minor product corresponded to coordinates 4978742..4979422 in the *Pst* DC3000 genome sequence. This region is more than 22 kb downstream from the 165 kb duplicated region and most likely formed as a result of mis-priming.

Whole genome sequencing was used to determine whether the 165 kb duplication was present in other *Pst* isolates. In addition to *Pst* DC3000(H0457), nine other *Pst* genomes were sequenced (Table 1). Seven of these isolates were derived from the *Pst* DC3000(H0457) either by subculture (Table 1, *Pst* DC3000(ATCC, PS1, JB, BB, ZB, LS) or by genetic manipulation (*Pst* DC3000(D36E) is a *Pst* DC3000(H0457) derivative in which 36 confirmed effectors have been deleted). *Pst* DC3000(JF) is an independently propagated *Pst* DC3000 and *Pst* NCPPB1106 is the progenitor of *Pst* DC3000 [26], [30]. Sequencing based copy number analysis indicated that the 165 kb duplication was present in five isolates. However, two isolates derived from *Pst* DC3000(H0457) by genetic manipulation (*Ps*t DC3000(D36E) or by *in vitro* culture (*Pst* DC3000(JB)) did not have the 165 kb duplication (Fig. S4). Additionally, the independently propagated *Pst* DC3000(JF) and the progenitor of *Pst* DC3000(*Pst* NCPPB1106) did not show any evidence of the 165 kb duplication. PCR was used to test for the presence of the duplication in the sequenced isolates (Fig. 4). In all cases except for one the PCR results were consistent with sequencing based copy number analysis. The exception was *Pst* DC3000(JB), which PCR indicated (data not shown) it carried the Class I genotype similar to *Pst* DC3000(H0457) in Fig. 4. This discrepancy may be due to bona fide loss of the duplication in the sequenced clone and not in the stored freezer stock or possibly due to a technical limitation of the copy number analysis method.

All isolates containing the duplication were identical over the entire 165 kb region, suggesting that the duplication was recently acquired. However, we did find an interesting polymorphism among the isolates that lack the 165 kb duplication. First, the near-effectorless deletion derivative, *Pst* DC3000(D36E), appears to lack the duplication, but retains a copy of ISPsy5 at each end of the 165 kb locus (Fig. 4). In comparison, *Pst* DC3000(JF) and *Pst* NCPPB1106 lack both the duplication and the left end ISPsy5 element. These polymorphisms were confirmed by Sanger sequencing PCR products that covered the left ISPsy5 integration site. Together, these results suggest that in the course of laboratory propagation, *Pst* DC3000(H0457) acquired an ISPsy5 insertion at the left end of the 165 kb region (*Pst* DC3000 genome coordinate 4790732) and that this IS element provided the homology that led to duplication of the 165 kb region. Additionally, the discovery of this polymorphism also allowed us to confirm the eight base target duplication generated upon ISPsy5 insertion, as mentioned above.

PCR experiments used to confirm the presence of the duplication were also used to test a sample of genomic DNA (provided by Robin Buell, Michigan State University) that was left over from the original *Pst* DC3000 sequencing effort reported by Buell et al., 2003 [29]. This PCR confirmed that the *Pst* DC3000 genomic DNA used to generate the reference sequence contained the 165 kb duplication (see DC3000(Buell) in Fig. 4B).

### Duplication conditionally affects growth

Genomic duplications are part of the evolutionary toolbox and can dramatically change a cell's phenotype. The magnitude and direction of the change depends on the costs and benefits associated with the characteristics of a specific rearrangement. For example, doubling the copy number of genes can increase expression and provide a benefit; or negatively impact fitness through increased metabolic burden associated with additional sequence replication/maintenance or replichore imbalance [47].

**Figure 5:**
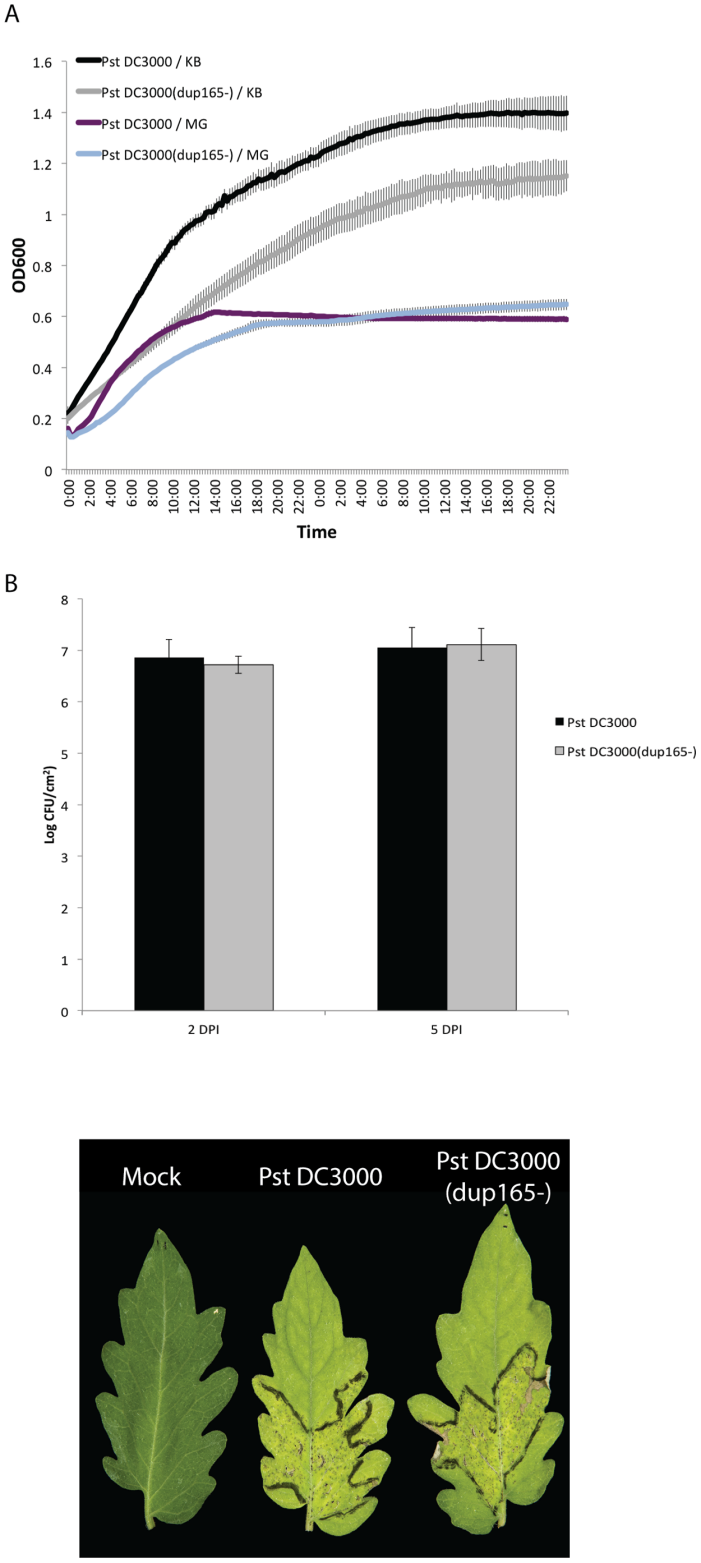
Phenotypes conferred by the 165 kb duplication. The growth of *Pst* DC3000 that contains the 165 kb duplication was compared to isogenic *Pst* DC3000(*dup165*-) cells with the duplication removed. A. Shows the phenotype of cells grown in rich (KB) and minimal (MG) media. B. 3×10^4^ cfu/ml of *Pst* DC3000 or *Pst* DC3000(*dup165*-) were infiltrated into four-five week old tomato (*Solanum lycopersicom* ‘Moneymaker’) plants. The number of bacterial cells per square centimeter of leaf tissue was determined at two and five days post inoculation. Leafs show disease symptoms at five days post inoculation.

To determine whether the 165 kb duplication affected the phenotype of *Pst* DC3000 cells, the majority (163,216 bp) of the left copy was deleted using recombineering to generate *Pst* DC3000(*dup165* ^−^) (Fig. S5). The growth of the *Pst* DC3000(*dup165* ^−^) deletion mutant was compared to *Pst* DC3000(H0457) in rich (KB) and minimal (MG) liquid growth media (Fig. 5A). Surprisingly, *Pst* DC3000 cells containing the duplication grew better than cells in which the duplication had been removed. The effect of the duplication on growth was most pronounced in rich medium, where duplication carrying cells grew approximately 44% faster in log phase (Fig. S1) and reached a higher final density before reaching the stationary phase. The improved growth of duplication containing cells was also detected in minimal medium, but only when the cells were in the log phase and growing at the fastest rate (Fig. 5A).

**Figure 6:**
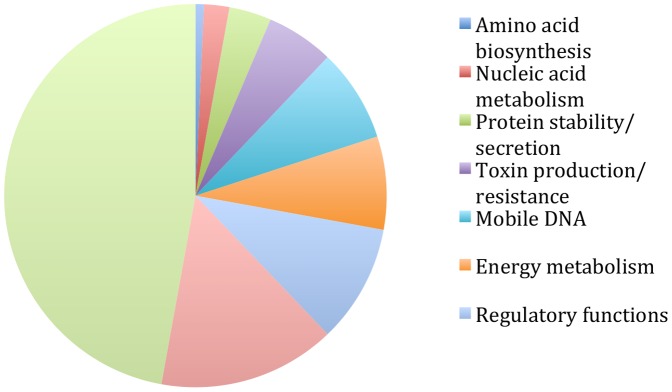
The functional role classification for genes in the 165 kb duplication. The functional roles for the 140 genes in the 165-Comprehensive Microbial Resource (JCVI-CMR) Cellular Role Category annotation [48]. The JCVI-CMR Cellular Role Category annotation for each gene in the duplication is shown in Table S2.

The 165 kb region contains 140 annotated protein coding genes. The functional roles [48] of each gene were reviewed to determine whether any might contribute to the growth phenotype or confer other important characteristics (Fig. 6 and Table S3). Overall, the range of functions encoded within the 165 kb region did not differ substantially from the genome-wide statistics (Fig. S6). Nevertheless, the annotation suggested that four genes within the 165 kb region had functions associated with pathogenesis; these are *gor-2*, *pnlA*, *sodB* and *hopE1* (Table S3). We tested whether the duplication influenced the ability of *Pst* DC3000 to function as a virulent pathogen on tomato. Plants were infiltrated with *Pst* DC3000(H547) or *Pst* DC3000(*dup165* ^−^); symptoms were observed over 5 days and the growth of the bacteria was assessed on days two and five post inoculation. No significant differences in disease or *in planta* growth were noted (Fig. 5B), suggesting that the growth defect observed *in vitro* does not affect the bacterial physiology with respect to its pathogenic lifestyle.

### Dynamic properties of the 165 kb duplication in *Pst* DC3000

**Figure 7:**
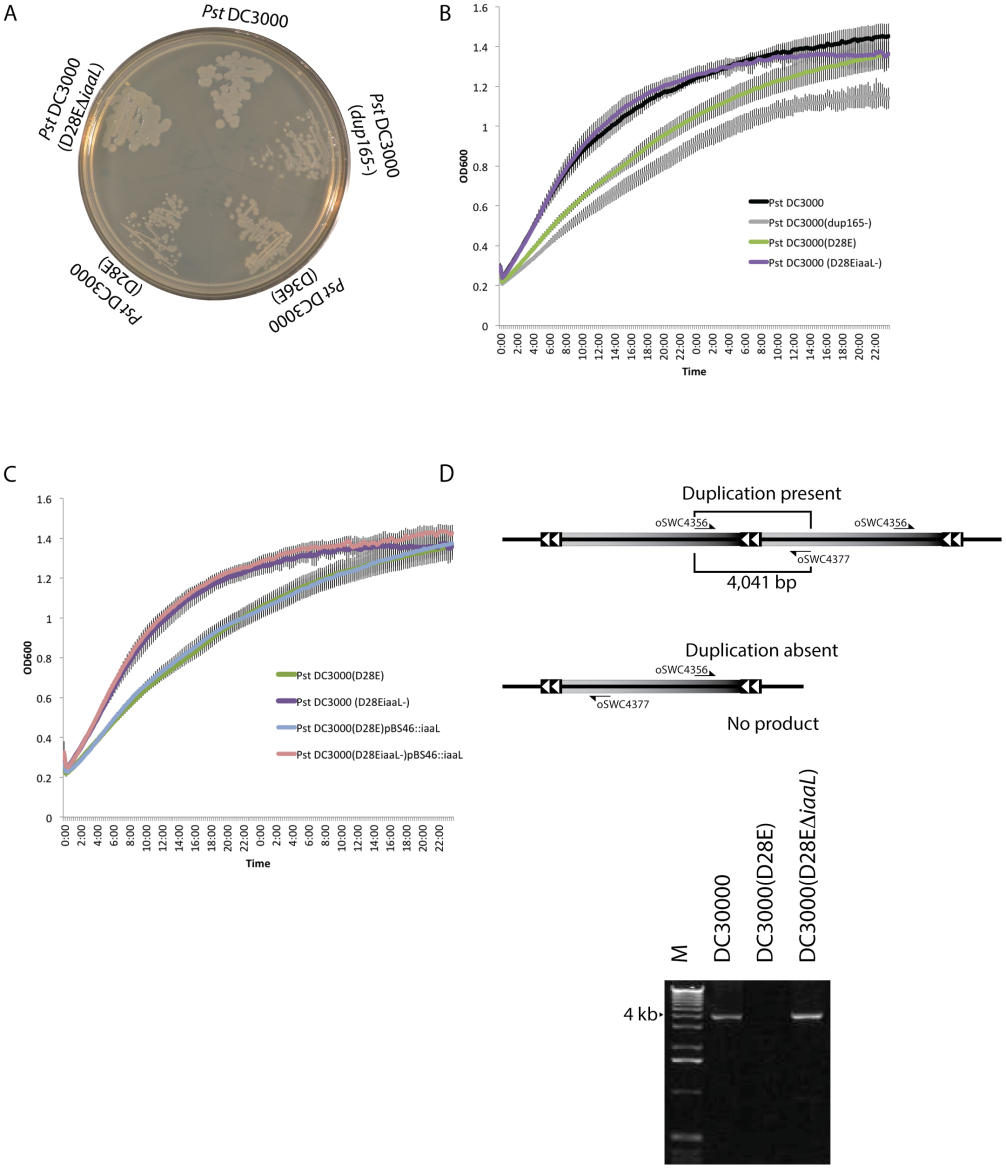
Presence of the 165 kb duplication fluctuates. A spontaneous mutant that had reformed the 165°C. B. The *Pst* DC3000(D28E **Δ** *iaaL*) mutant had an increased growth rate in rich (KB) broth medium. C. Expression of the *iaaL* gene in trans does not complement growth phenotype of the *Pst* DC3000(D28E**Δ** *iaaL*) mutant. D. The duplication was present in *Pst* DC3000(D28E**Δ** *iaaL*), but not in the parental *Pst* DC3000(D28E) strain. PCR with primers oSWC4356 and oSWC4377 produce a 4041 bp product when the duplication is present. No product is formed when the duplication is absent. Lane marked ‘M’ indicates molecular weight marker.

As noted above, we observed that the 165 kb duplication was absent in some *Pst* DC3000(H0457) derivatives i.e., see *Pst* DC3000(D36E) in Fig. 4. Since these types of duplications are known to be unstable [31], it would not be surprising if some other *Pst* DC3000 mutants differ with respect to the presence of the duplication. Consistent with this idea, we observed the growth phenotype of a *Pst* DC3000 mutant change after a routine genetic manipulation using allelic exchange mutagenesis to delete a gene. In this case, the *iaaL* gene was deleted from *Pst* DC3000(D28E). The deletion strain consistently yielded larger colonies than the *Pst* DC3000(D28E) parent on KB agar plates (Fig. 7A). The difference in growth was confirmed using a growth curve in liquid KB broth (Fig. 7B). Furthermore, expression of the *iaaL* gene *in trans* failed to complement this phenotype (Fig. 7C), suggesting that absence of *iaaL* was not responsible for the altered growth. PCR confirmed that *Pst* DC3000(D28E) and the *iaaL* mutant strains differ in their duplication status (Fig. 7D). Because the constructed duplication deletion mutant, *Pst* DC3000(*dup165* ^−^) (Fig. 5 and Fig. 7) also exhibits slow growth phenotype in rich medium, this finding provides a second example linking the growth phenotype with the duplication and raises the possibility that there is positive selection for the duplication in cells growing under laboratory conditions.

## Discussion

Genomes are the fundamental structural and operational unit of life, upon which all genetic and regulatory information are organized, utilized and stored. Genomes also have adaptive functions that allow evolution to proceed by the capacity to balance stability with change. The pace and targets of change are influenced by genomic organization, suggesting that the flexibility we observe is the product of many successful evolutionary steps. In bacteria, genome fluidity is mechanistically linked to horizontal gene transfer, mutation and intrachromosomal recombination. Here we describe an example of genome fluidity mediated by the latter and show that IS elements can contribute to this process by serving as segments of mobile homology that can facilitate recombination. IS-mediated duplications are a useful evolutionary device because they occur readily, usually do not cause loss of function, and can collapse back to single copy if a selective advantage is not produced; an evolutionary gamble with very low stakes. In the long term, duplications serve as the birthplace for novel functions by providing additional sequences that can accumulate mutations and allow diversification without compromising essential activities [24].

### Identifying the 165 Kb duplication in *Pst* DC3000

The *Pst* DC3000 genome sequence was reported in 2003 [29] and was later resequenced as a proof of concept for genome assembly using short read sequence data [49]. It is not surprising that neither report described the presence of the 165 kb duplication, because the ISPsy5 insertion sequence elements that flank the region complicate identification and mapping strategies. IS elements and other repetitive sequences, that are too long to be spanned by a continuous sequence read, make tandem duplications impossible to directly map. In general, short read sequences derived from any repetitive DNA element that cannot be spanned by a single read are considered “unmappable” and their location must be inferred by the unique sequence joints at the 5′ and 3′ ends of the repetitive element. We detected the presence of the duplication in Illumina short read sequencing using profiles showing read coverage for the entire genome; similar observations were described in *Salmonella* [50]. To quantify the change in copy number, we developed a computational strategy that compensated for the *ori*-*ter* replication gradient. This approach implements ideas presented by Skogaard et al., 2011 [41] and should have utility for identifying other large-scale changes that alter genome structure.

The copy number analysis indicated that a 165 kb region had doubled. The structure of the duplication was probed using PCR and recombineering, and further verified with traditional Sanger sequencing. The duplicated region accounts for 2.6% of the total genome and contains 140 annotated protein-coding genes, including the *hopE1* virulence effector gene [51], [52]. The duplication is flanked by 2059 bp ISPsy5 elements. We note that there are 33 complete copies of the ISPsy5 element in the *Pst* DC3000 reference sequence which could facilitate other genomic rearrangements.

### The 165 kb duplication formed in domesticated *Pst* DC3000 isolates

The *Pst* DC3000 strain became a model bacterium largely because of pioneering work that demonstrated this strain was capable of DNA uptake (transformation and conjugation) [30] and could cause disease on *Arabidopsis* [53]. The *Pst* DC3000 strain was isolated in the mid-1980's as a spontaneous rifampicin resistant mutant of *Pst* NCPPB1106, a feature that was used to select for conjugal recipient clones [26], [30]. It was at this point, that the evolutionary trajectories of the two strains diverged. We analyzed *Pst* NCPPB1106 for the presence of the 165 kb duplication to determine if it had been present before *Pst* DC3000 was isolated and therefore likely to be propagated to all *Pst* DC3000 strains and derivatives. We found that *Pst* NCPPB1106 did not have the duplication or the ISPsy5 insertion and target site duplication that marks the left boundary of the duplicated region. Because the IS elements likely provide the necessary homologies for formation of the tandem duplication, the lack of the ISPsy5 insertion at this location is strong evidence that *Pst* NCPPB1106 has never had the 165 kb duplication. It also implies that not all *Pst* DC3000 isolates necessarily contain the duplication or the homology needed for it to form. Consistent with this hypothesis a *Pst* DC3000 isolate obtained in 2012 from Jacqueline Fletcher at Oklahoma State University contains the *Pst* NCPPB1106 allele (see *Pst* DC3000(JF) in Fig. 4) confirming that at least one *Pst* DC3000 isolate did not inherit the duplication.

The absence of the left end ISPsy5 in *Pst* NCPPB1106 suggests that the 165 kb duplication in *Pst* DC3000 likely formed in two steps since these strains diverged. First, an ISPsy5 element inserted at *Pst* DC3000 genome coordinate 4790732, which forms the left boundary of the affected region and introduced the homology necessary for the next step. Second, a recombination event occurred between the IS elements at the left and right boundaries, likely by an unequal crossing over, that resulted in formation of the tandem duplication.

### The duplication confers a selective advantage in some conditions

Given our current understanding of the relationship between genes, copy number and genomic organization, it is very difficult to predict whether a duplication will have phenotypic effects, especially in terms of any emergent characteristics that might arise from the interplay between these features. Even so, duplications can alter the phenotype of cells that contain these structures (for review see [54], [55]). We found that the 165 kb duplication affected the phenotype of *Pst* DC3000 in conditions that support rapid growth. Under these conditions, the duplication-containing *Pst* DC3000(H0457) strain grew more vigorously and to a higher final density compared to the mutant lacking the duplication (Fig. 5A).

It is important to note that we did not detect any difference in disease or *in planta* growth associated with the 165 kb duplication (Fig. 5B), suggesting that any variability in the duplication status is unlikely to effect analysis of *P. syringae* virulence systems. Additionally, this result provides information about *Pst* DC3000 *in planta* growth dynamics. Because we did not see any difference in *in planta* growth between cells that differed in duplication status, this supports the idea that the apoplastic environment is more similar to minimal media than rich broth conditions [56], and that cells grow relatively slowly *in planta* compared to in rich broth.

In general, finding a persistent duplication can be taken as evidence of positive selection. Based on our phenotypic observation, it's likely that the duplication has become fixed in some *Pst* DC3000 strains because of the fitness advantage these cells possess in rich culture. We do not yet have a mechanistic explanation for how the duplication enables faster growth. We found no evidence of SNPs between the duplicated copies in the whole genome sequence data, suggesting that the duplication was recently acquired and that any observed phenotypic affects were unlikely a result of gene-level functional adaptation. Therefore, it seems that the growth phenotype is either a result of increased copy number or altered genomic organization. Gene copy number amplification has been proposed to explain the now classic (and provocative) results of Cairns and Foster [57], who found *lac* ^−^ revertants able to grow on lactose at a frequency higher than could be explained by the normal mutation rate. The amplification mutagenesis model holds that duplication and higher order gene amplification can enable cells to survive nonpermissive growth conditions by providing additional copies of genes that are weakly beneficial in that environment [58]. Similar biological phenomena are likely in operation with the 165 kb duplication. There are 140 genes encoded in this duplicated region and it is possible that increased expression of one or more of them enables cells to grow better than single copy peers in rich media conditions.

### Flexibility and stability

It did not escape our attention that the altered *in vitro* growth characteristics of the duplication deletion *Pst* DC3000(*dup165* ^−^) were similar to the growth phenotype of the near-effectorless *Pst* DC3000(D36E) mutant. The growth phenotype noted in the *Pst* DC3000(D36E) strain arose spontaneously in several independent mutant lines generated in the course of producing the near-effectorless mutants, i.e., *Pst* DC3000(D28E) [33]. The altered growth phenotype of the effector polymutant strains was initially observed as colonies that were markedly smaller when plated on KB (rich) agar medium (Fig. 7A) and grew to the same size as wild type on minimal (MG) agar growth medium [33]. This led us to hypothesize that the growth defect of the *Pst* DC3000 multiple effector mutant strains was attributed to the status of the duplication. This hypothesis was supported by two observations. First, *Pst* DC3000(D36E) and an earlier deletion derivative *Pst* DC3000(D28E) (in which the 28 most highly expressed effectors have been deleted) lacked the duplication and did not grow as well in rich medium as strains containing the 165 kb duplication (Fig. 7). Second, in the course of a routine genetic manipulation (deletion of *iaaL* from *Pst* DC3000(D28E), see material and methods) we observed a spontaneous reversion to the enhanced growth phenotype (Fig. 7) and when we assayed for the presence of the duplication using PCR we found that the duplication had returned (Fig. 7D). Expression of the deleted *iaaL* gene from a plasmid did not complement, supporting the notion that it is not responsible for the growth phenotype (Fig. 7C). The identification of strains in which the 165 kb duplication spontaneously fluctuates demonstrates the dynamic nature of these sorts of genomic rearrangements and provides further evidence implicating the duplication with the growth phenotype.

Tandem duplications are notoriously unstable. Formation, loss and amplification of this type of duplication are predicted to occur at high frequency [31]. In *Salmonella typhimurium*, the frequency of duplication at most genomic loci is between 10^−3^ and 10^−4^ [59]. There is also a high rate of loss, which led to the proposal that the observed presence of duplications in a population represents a steady state. In an unselected population, its estimated that approximately 1 cell in 1000 contains a duplication [60]. This implies that the *Pst* DC3000 genome experiences a certain degree of flux and that what we observe (either physically or phenotypically, i.e., via sequencing or genetics) represents a steady-state equilibrium of the analyzed population.

### Relevance to *P. syringae* biology

Speculation regarding the role of IS elements in promoting recombination in *P. syringae* began shortly after repetitive sequences were found in association with the first avirulence gene cloned from these bacteria [61]. Since then, many examples of *P. syringae* virulence genes located near or in association with mobile genetic elements have been found and the idea that these elements might facilitate recombination has been a recurring theme [22], [62], [63]. Moreover, comparison to diverse *P. syringae* strains suggests that members of the *Pst* DC3000 phylogenetic clade have evolved to use transposons to generate diversity more than other *P. syringae* groups [17], [64]. It is striking that 5% of *Pst* DC3000 ORFs are annotated as having functions related to transposition [29]. Additionally, there is evidence of recent transposon activity in *Pst* DC3000 that altered genomic content of some laboratory isolates [21]. These characteristics and the results described here suggest the *Pst* DC3000 genome has some structural fluctuation, which in some cases may provide advantages in certain environments.

## Supporting Information

Figure S1 **Analysis of growth rate data for** ***Pst*** **DC3000 and** ***Pst*** **DC3000 (** ***dup165*** **^−^) grown in KB medium.** Main plot: Approximate instantaneous growth rates for each strain, calculated by numerically differentiating the log-transformed growth curves log_2_(N/N_0_) vs t. Growth rates are reported as an inverse doubling time (hr^−1^). Inset plot: Estimated ratio of *Pst* DC3000 to *Pst* DC3000(*dup165* ^−^) growth rates, using both the unsmoothed data shown in the main plot (black), and smoothed versions of the growth rate data (red), doing the smoothing with a boxcar filter of width 10. From the inset, it can be seen that during the first 6 hours, *Pst* DC3000 grows at a rate approximately 44% times faster than *Pst* DC3000(*dup165* ^−^).
(TIF)

Figure S2 **ChIP-seq experiments revealed the presence of genomic rearrangements.** Illumina sequencing results shown are from control samples used in ChIP-seq experiments to identify iron starvation sigma factor binding locations. The number of sequence reads (Y-axis) for each position of the genome (X-axis) is shown as a histogram. These results indicate that the region spanning 4790778..4955784 in the annotated *Pst* DC3000 sequence had approximately twice the local average number of sequence reads. The data shown here were described as negative controls in ChIP-seq experiments analyzing the regulons iron starvation (IS) sigma factors [1], [2]. In these IS sigma factor studies, enrichment and non-specific immunoprecipitation were evaluated by comparing the number of sequence reads at each locus in experimental samples versus reads from controls prepared from *Pst* DC3000 cells containing the empty vector (pBS60). This control was treated identically to cells expressing the epitope tagged sigma factor [1], [2]. The duplicated DNA sequence is visible as doubled DNA content near 5e+06 bp. The partition of the *Pst* DC3000 replichores was also suggested in the ChIP-seq results. For example, we observed that genomic DNA proximal to the presumed origin of replication was more abundant relative to parts of the chromosome where replication is expected to terminate. This is likely due to the presence of multiple replication forks proceeding bidirectionally from the origin of replication [4], [5]. Red and green histograms show the number of sequence reads matching the positive and negative strands of the *Pst* DC3000 genome sequence [3], respectively.
(TIF)

Figure S3 **ISPsy5 inverted repeats.** Boundaries of the ISPsy5 elements were identified by searching the *Pst* DC3000 genome sequence using Blast with a representative ISPsy5-containing sequence as the query. The 5′ ends of each end were aligned and internal boundary of the inverted repeats was determined as the point where the sequence identity between the two inverted repeats fell below 80% using a 10 bp sliding window.
(TIF)

Figure S4 **Copy number analysis of** ***Pst*** **genome sequences.** Whole genome assisted copy number analysis was used to determine whether genome sequences show evidence of genomic duplication. *Pst* DC3000(JF) does not have the 165 kb duplication, but shows evidence of another genomic expansion at 5369001..5402000, which is also flanked by ISPsy5 elements.
(TIF)

Figure S5 **The left hand (terminus proximal) copy of the duplication was deleted.** The genome coordinates of this deletion are 4790396..4953611. Recombineering was used to delete 163,216 bp of the left copy of the duplication and replaced with the kanamycin resistance encoding *neo* gene. PCR and sequencing were used to confirm deletion of the duplication. PCR products were resolved on a 1% agarose gel. M, molecular weight marker.
(TIF)

Figure S6 **Functional roles of genes within the duplication do not differ substantially from the genome-wide proportions.** The functional role categories for the *Pst* DC3000 genome were obtained from JCVI Comprehensive Microbial Resource database [6]. The fractional representations of genes from each category are shown for the entire genome (all) in blue and for the 165 kb duplication (dup) in green.
(TIF)

Table S1 **Oligonucleotides used.**
(DOCX)

Table S2 **Areas of the** ***Pst*** **DC3000(ATCC) genome with altered copy number.** Illumina sequencing results for *Pst* DC3000(ATCC) genome were analyzed to identify genomic regions with anomalous read depth coverage. The relative read depth of 1,000 bp genome sequence blocks was determined in relation to the expected local average. The expected local average was computed across the genome in order to adjust for differences in read depth due to the replication-to-terminus sequence content gradient. The average relative read depth coverage for the entire genome was approximately one (log_2_ mean = −0.02 stddev = 0.23). Windows of 1,000 bp with relative read depth coverage that differed by more than three standard deviation from the local average are shown above. Relative sequence read depth is a log_2_-transformed measure of read depth (X), retransformed back to the linear space (2^X^) to produce the Estimated copy number. Regions with doubled relative sequence coverage do not map exactly with the predicted duplicated region because the resolution of this copy number analysis is lower due to the requirement that the entire 1000 bp region have an average that is more than three standard deviations from the expected local average and because each end of the duplicated region terminates with unmappable ISPsy5 elements.
(DOCX)

Table S3 **Genes and functional role categories contained within the 165 kb duplication.** Multiple JCVI categories are separated by a “/”. In instances where a gene does not have a JCVI category assigned, the primary genbank annotation is shown. Genes annotated as having roles in pathogenesis are indicated with “♦”. Genes in the same functional role category are indicated by the color of background shading. *Disrupted reading frame. ** No JCVI Cellular role category information.
(DOCX)
