# Supplementary figures and images for: Genomic Plasticity Enables Phenotypic Variation of Pseudomonas syringae pv. tomato DC3000
Source: PLoS One. 2014 Feb 6;9(2):e86628. doi: 10.1371/journal.pone.0086628 (PMC3916326; doi:10.1371/journal.pone.0086628)

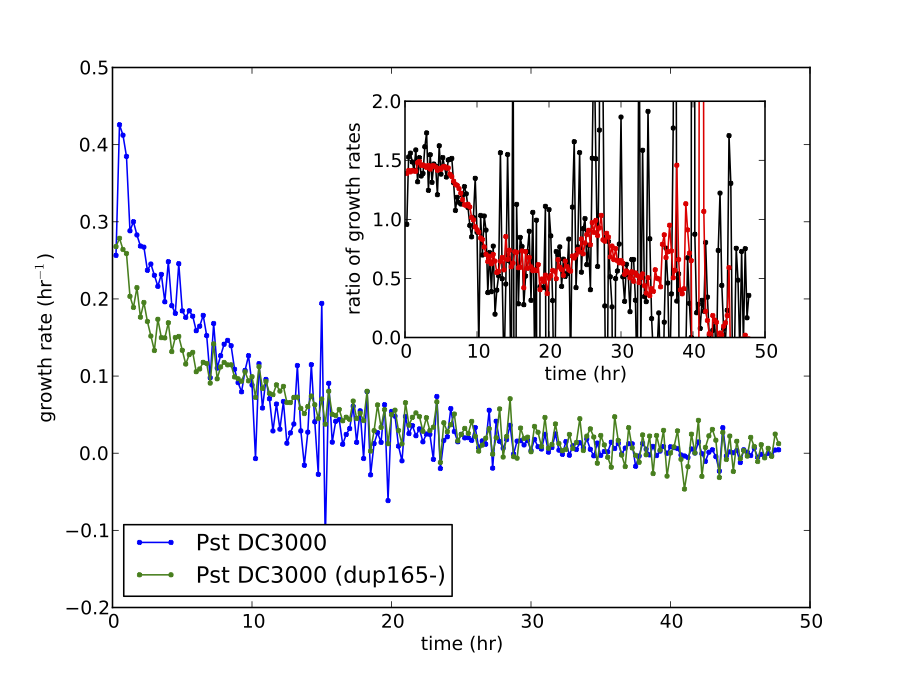

Supplement: Figure S1 — Analysis of growth rate data for Pst DC3000 and Pst DC3000 ( dup165 −) grown in KB medium. Main plot: Approximate instantaneous growth rates for each strain, calculated by numerically differentiating the log-transformed growth curves log2(N/N0) vs t. Growth rates are reported as an inverse doubling time (hr−1). Inset plot: Estimated ratio of Pst DC3000 to Pst DC3000(dup165 −) growth rates, using both the unsmoothed data shown in the main plot (black), and smoothed versions of the growth rate data (red), doing the smoothing with a boxcar filter of width 10. From the inset, it can be seen that during the first 6 hours, Pst DC3000 grows at a rate approximately 44% times faster than Pst DC3000(dup165 −). (TIF) [file pone.0086628.s001.tif]

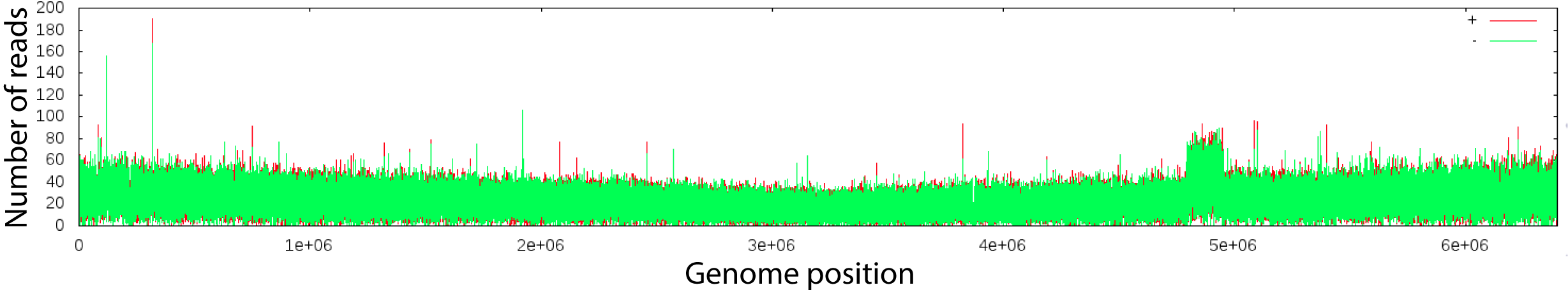

Supplement: Figure S2 — ChIP-seq experiments revealed the presence of genomic rearrangements. Illumina sequencing results shown are from control samples used in ChIP-seq experiments to identify iron starvation sigma factor binding locations. The number of sequence reads (Y-axis) for each position of the genome (X-axis) is shown as a histogram. These results indicate that the region spanning 4790778..4955784 in the annotated Pst DC3000 sequence had approximately twice the local average number of sequence reads. The data shown here were described as negative controls in ChIP-seq experiments analyzing the regulons iron starvation (IS) sigma factors [1], [2]. In these IS sigma factor studies, enrichment and non-specific immunoprecipitation were evaluated by comparing the number of sequence reads at each locus in experimental samples versus reads from controls prepared from Pst DC3000 cells containing the empty vector (pBS60). This control was treated identically to cells expressing the epitope tagged sigma factor [1], [2]. The duplicated DNA sequence is visible as doubled DNA content near 5e+06 bp. The partition of the Pst DC3000 replichores was also suggested in the ChIP-seq results. For example, we observed that genomic DNA proximal to the presumed origin of replication was more abundant relative to parts of the chromosome where replication is expected to terminate. This is likely due to the presence of multiple replication forks proceeding bidirectionally from the origin of replication [4], [5]. Red and green histograms show the number of sequence reads matching the positive and negative strands of the Pst DC3000 genome sequence [3], respectively. (TIF) [file pone.0086628.s002.tif]

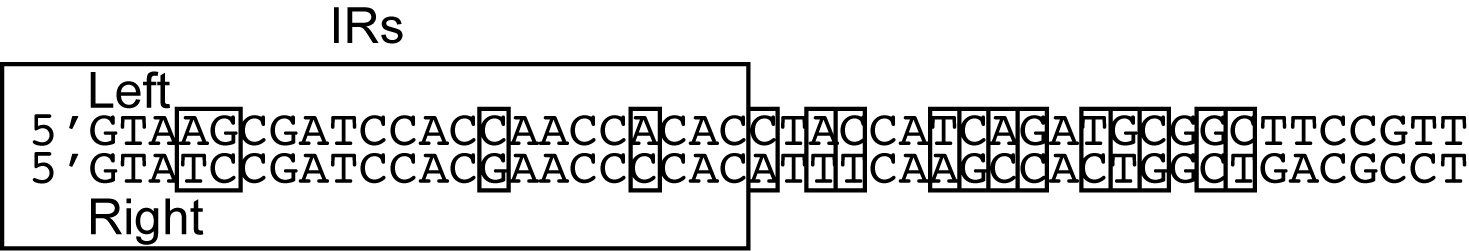

Supplement: Figure S3 — ISPsy5 inverted repeats. Boundaries of the ISPsy5 elements were identified by searching the Pst DC3000 genome sequence using Blast with a representative ISPsy5-containing sequence as the query. The 5′ ends of each end were aligned and internal boundary of the inverted repeats was determined as the point where the sequence identity between the two inverted repeats fell below 80% using a 10 bp sliding window. (TIF) [file pone.0086628.s003.tif]

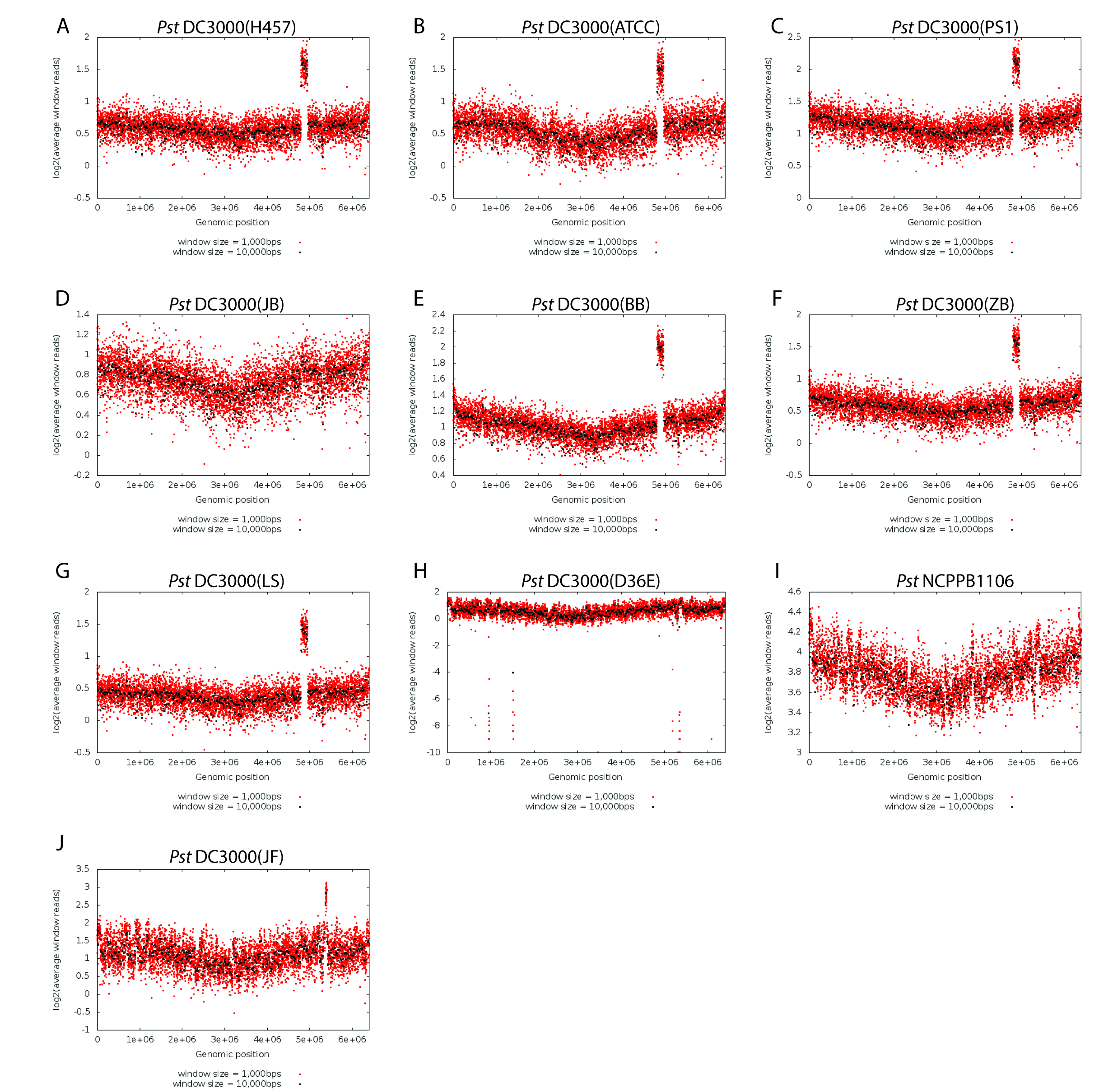

Supplement: Figure S4 — Copy number analysis of Pst genome sequences. Whole genome assisted copy number analysis was used to determine whether genome sequences show evidence of genomic duplication. Pst DC3000(JF) does not have the 165 kb duplication, but shows evidence of another genomic expansion at 5369001..5402000, which is also flanked by ISPsy5 elements. (TIF) [file pone.0086628.s004.tif]

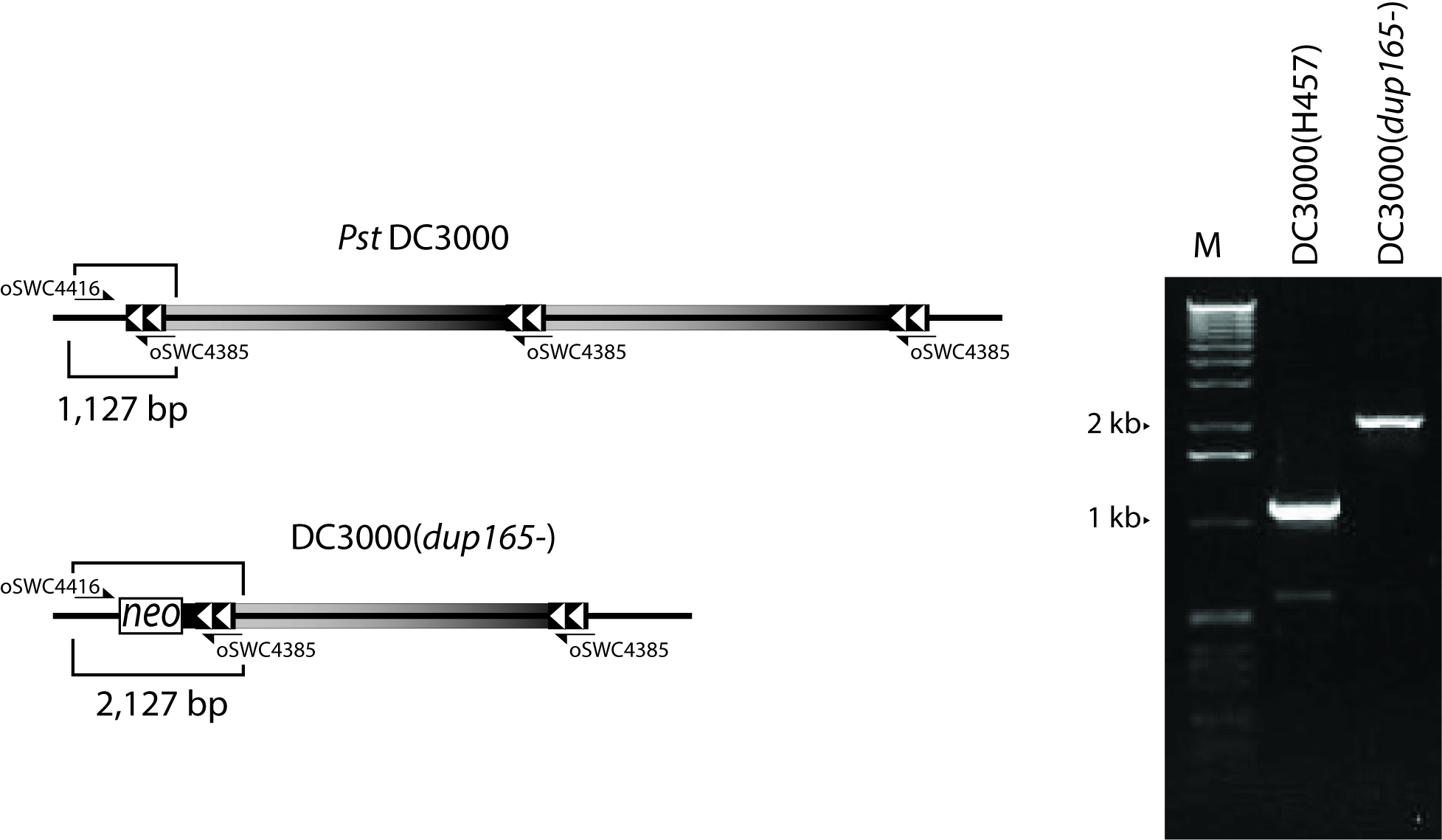

Supplement: Figure S5 — The left hand (terminus proximal) copy of the duplication was deleted. The genome coordinates of this deletion are 4790396..4953611. Recombineering was used to delete 163,216 bp of the left copy of the duplication and replaced with the kanamycin resistance encoding neo gene. PCR and sequencing were used to confirm deletion of the duplication. PCR products were resolved on a 1% agarose gel. M, molecular weight marker. (TIF) [file pone.0086628.s005.tif]

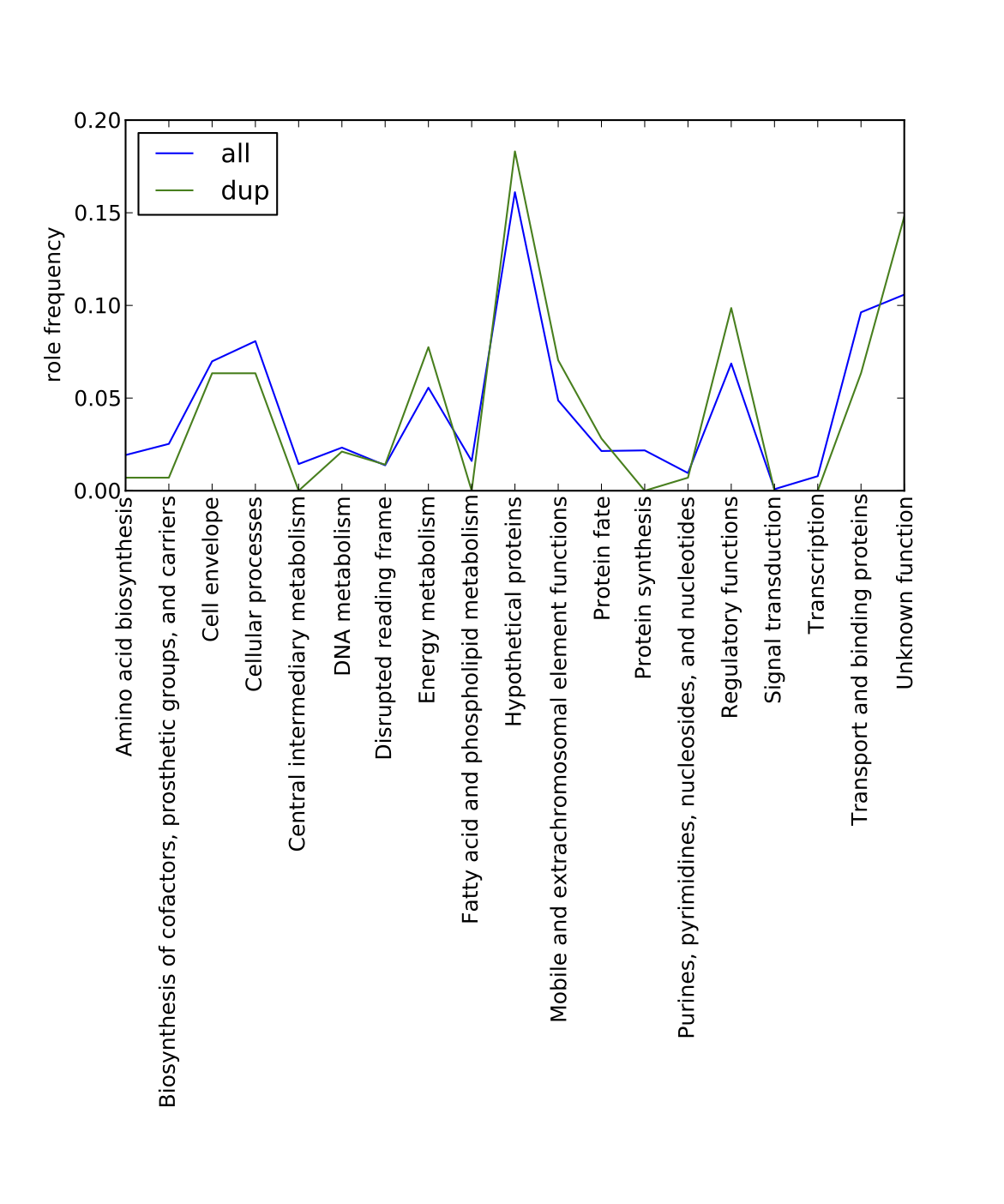

Supplement: Figure S6 — Functional roles of genes within the duplication do not differ substantially from the genome-wide proportions. The functional role categories for the Pst DC3000 genome were obtained from JCVI Comprehensive Microbial Resource database [6]. The fractional representations of genes from each category are shown for the entire genome (all) in blue and for the 165 kb duplication (dup) in green. (TIF) [file pone.0086628.s006.tif]
