# Supplementary material for: Genomic Plasticity Enables Phenotypic Variation of Pseudomonas syringae pv. tomato DC3000
Source: PLoS One. 2014 Feb 6;9(2):e86628. doi: 10.1371/journal.pone.0086628 (PMC3916326; doi:10.1371/journal.pone.0086628)
Supplement: Table S1 — Oligonucleotides used. (DOCX) [file pone.0086628.s007.docx]

| Oligo | Description | Sequence (5’→3’) |
| --- | --- | --- |
| HL117 | Left flanking region for PSPTO_0371 deletion | ATTATCTAGACATCATTGCCACTGTCTGTTGCG |
| HL118 | Left flanking region for PSPTO_0371 deletion | CCATTCCTTTTCTACATCGTAGGCAGTCA |
| HL119 | Right flanking region for PSPTO_0371 deletion | TACGATGTAGAAAAGGAATGGATCATTGACCGCAGGAATTGAAT |
| HL120 | Right flanking region for PSPTO_0371 deletion | ATTATCTAGAGTATTACGCAAGTGCTTCAATCGCAATC |
| HL121 | PCR screen for PSPTO_0371 deletion construct; sequencing mutant allele | GTTCTGTCGATGGGGCGGTCTTC |
| HL122 | PCR screen for PSPTO_0371 deletion construct; sequencing mutant allele | AGGTGGCATGTGCTTTAGCGAACAAG |
| HL123 | Sequencing PSPTO_0371 deletion | CATCGCCGTGGCAGTCTCTTCA |
| HL124 | Sequencing PSPTO_0371 deletion | TGTCTACACTTTCCGGGTTTCTCTCTAAC |
| HL161 | Sequencing PSPTO_0371 deletion | CGTGTCGCCCGCCTGACCTG |
| HL162 | Sequencing PSPTO_0371 deletion | GTTCGGAATTGCCTTGCCAGC |
| oSWC04356 | 4952135-4952158; Pfd1; PCR/sequencing for duplication test | TCCAAGTTGGGTGCCTCAGGCTTG |
| oSWC04357 | 4956364-4956342; Pfd1 | AGTAATCGTGTGTGCGGCACGAG |
| oSWC04377 | 4793099-4793079; Prd20; PCR/sequencing for duplication test | TTCACTACGACCAGACCAGGG |
| oSWC04378 | 4790376-4790395; Prd20cf | TCCTGCTGAATCTACGCAGG |
| oSWC04379 | 4952690-4952669; Prd20seq1 | TTAGGAGCTCTACCGCCTCGTC |
| oSWC04380 | 4952572-4952593; Prd20seq2 | TGGCGCGCTTGACTCTCTAAAG |
| oSWC04381 | 4953204-4953185; Prd20seq3 | TTGAACGCGGGGTACCATTG |
| oSWC04382 | 4953076-4953097; Prd20seq4 | TCGGCAGGATATCGACCAATGG |
| oSWC04383 | 4953627-4953607; Prd20seq5 | TGCGATCTTCGCCGCCTTCAC |
| oSWC04384 | 4953519-4953540; Prd20seq6 | ACACGCTTTGGTATTGGTCGGG |
| oSWC04385 | ISPsy5 l; Prd20seq7; PCR/sequencing for duplication test | TGTTCAGCGACACGCCTAAAGG |
| oSWC04386 | ISPsy5; Prd20seq8 | AGCCACGTATAGGGCTCCTG |
| oSWC04387 | ISPsy5; Prd20seq9 | TCGCGGCTATGTGATGACTG |
| oSWC04388 | ISPsy5; Prd20seq10 | ACGCCCGGTCTTGCCCTTAGGTTG |
| oSWC04389 | ISPsy5; Prd20seq11 | AAAAGAGCATGGCCAGCCCCAG |
| oSWC04390 | ISPsy5; Prd20seq12 | TGCCATGTCGGCTTAGCACC |
| oSWC04391 | ISPsy5; Prd20seq13 | ACGCCACTCACATCGTTGATCTC |
| oSWC04392 | ISPsy5; Prd20seq14 | AGGCTCGACGGTTTGTTCGG |
| oSWC04393 | ISPsy5; Prd20seq15 | TCCATCGATGGCTTAGCTG |
| oSWC04394 | ISPsy5; Prd20seq16 | AATCTTCACTCGATTCCGGG |
| oSWC04410 | PCR recombineering substrate; deletion/neo insertion at central junction | AAGCACCGTGGCATGATACCCACTTGCAGGGAGAAGAAGGAAACTATGTAGGCTATTGGGCTTTTGAGGCGGGGGCAATCTGCCGCAAGCACTCAGGGCGCAAGG |
| oSWC04411 | PCR recombineering substrate; deletion/neo insertion at central junction | AGCGCCCAGCGCCTGCCGTTGCGACAGAGCCGGGCGCCCAGTGCCAAAAGATCGATTAGCTCCCGACGGGCGTGCGCATGGTTACTCAGAAGAACTCGTCAAGAAGGCG |
| oSWC04412 | 4796397-4796376; PCR/sequencing duplication deletion | TTGCATGGGATTCTCACATGGG |
| oSWC04413 | 4952844-4952863; PCR/sequencing duplication deletion | AGTATGAAGTACGGCAGCTC |
| oSWC04414 | PCR recombineering substrate; deletion/neo insertion left 165 kb copy | TCTTGCACCTCACCTGGCAGACGCCTACGGCTTAGCTTTACAGTGCGCTCGAAACGCGCATCCTGCTGAATCTACGCAGGTGCCGCAAGCACTCAGGGCGCAAGG |
| oSWC04415 | PCR recombineering substrate; deletion/neo insertion left 165 kb copy | AGGCCGTCGTCCGGTCCTGGAATCCAGCCGATGACGGCGTTGACCGAATAGCGCATCACTGAGAGCGCTGCGATCTTCGCCGCCTCAGAAGAACTCGTCAAGAAGGCG |
| oSWC04416 | 4789842-4789863; PCR/sequencing for duplication test | TGTGACAGACACCTGTTCGGTG |
