# Supplementary material for: Genomic Plasticity Enables Phenotypic Variation of Pseudomonas syringae pv. tomato DC3000
Source: PLoS One. 2014 Feb 6;9(2):e86628. doi: 10.1371/journal.pone.0086628 (PMC3916326; doi:10.1371/journal.pone.0086628)
Supplement: Table S2 — Areas of the Pst DC3000(ATCC) genome with altered copy number. Illumina sequencing results for Pst DC3000(ATCC) genome were analyzed to identify genomic regions with anomalous read depth coverage. The relative read depth of 1,000 bp genome sequence blocks was determined in relation to the expected local average. The expected local average was computed across the genome in order to adjust for differences in read depth due to the replication-to-terminus sequence content gradient. The average relative read depth coverage for the entire genome was approximately one (log2 mean = −0.02 stddev = 0.23). Windows of 1,000 bp with relative read depth coverage that differed by more than three standard deviation from the local average are shown above. Relative sequence read depth is a log2-transformed measure of read depth (X), retransformed back to the linear space (2X) to produce the Estimated copy number. Regions with doubled relative sequence coverage do not map exactly with the predicted duplicated region because the resolution of this copy number analysis is lower due to the requirement that the entire 1000 bp region have an average that is more than three standard deviations from the expected local average and because each end of the duplicated region terminates with unmappable ISPsy5 elements. (DOCX) [file pone.0086628.s008.docx]

| Genomic coordinates | Relative sequence read depth (log_2_) | Estimated copy number |
| --- | --- | --- |
| 228001..229000 | -0.690525 | 0.619628325 |
| 4795001..4796000 | 1.097862 | 2.140372651 |
| 4796001..4797000 | 0.874281 | 1.833094294 |
| 4797001..4798000 | 1.137996 | 2.200751117 |
| 4798001..4799000 | 0.772036 | 1.70767804 |
| 4799001..4800000 | 1.097506 | 2.139844557 |
| 4800001..4801000 | 0.834383 | 1.783094297 |
| 4801001..4802000 | 0.68668 | 1.60957522 |
| 4802001..4803000 | 0.948261 | 1.929545414 |
| 4803001..4804000 | 1.03288 | 2.046104742 |
| 4804001..4805000 | 1.038296 | 2.05380043 |
| 4805001..4806000 | 1.06542 | 2.092779045 |
| 4806001..4807000 | 0.988751 | 1.984466214 |
| 4810001..4811000 | 1.171891 | 2.253068224 |
| 4811001..4812000 | 0.790033 | 1.729114014 |
| 4812001..4813000 | 0.649011 | 1.568092864 |
| 4813001..4814000 | 1.024619 | 2.034422045 |
| 4814001..4815000 | 1.133918 | 2.194539138 |
| 4815001..4816000 | 0.955368 | 1.939074186 |
| 4816001..4817000 | 0.974534 | 1.965006385 |
| 4817001..4818000 | 0.971094 | 1.960326553 |
| 4818001..4819000 | 0.993359 | 1.990814776 |
| 4819001..4820000 | 0.890596 | 1.853941858 |
| 4820001..4821000 | 1.123502 | 2.178752017 |
| 4821001..4822000 | 1.09203 | 2.1317378 |
| 4822001..4823000 | 0.96633 | 1.953863931 |
| 4823001..4824000 | 1.215104 | 2.321575179 |
| 4824001..4825000 | 1.023641 | 2.033043381 |
| 4825001..4826000 | 1.081059 | 2.115588444 |
| 4826001..4827000 | 1.139687 | 2.203332156 |
| 4827001..4828000 | 0.776602 | 1.713091249 |
| 4828001..4829000 | 1.079905 | 2.113896879 |
| 4829001..4830000 | 0.978153 | 1.96994179 |
| 4830001..4831000 | 1.180092 | 2.265912262 |
| 4831001..4832000 | 1.13839 | 2.201352224 |
| 4832001..4833000 | 1.074655 | 2.10621834 |
| 4833001..4834000 | 1.032051 | 2.044929349 |
| 4834001..4835000 | 0.80957 | 1.75268897 |
| 4835001..4836000 | 0.904296 | 1.871630962 |
| 4836001..4837000 | 0.835397 | 1.784347987 |
| 4837001..4838000 | 0.894054 | 1.858390907 |
| 4838001..4839000 | 0.860246 | 1.815347827 |
| 4839001..4840000 | 0.986291 | 1.9810853 |
| 4840001..4841000 | 1.08637 | 2.123390922 |
| 4841001..4842000 | 0.980894 | 1.973688072 |
| 4843001..4844000 | 1.160589 | 2.235486758 |
| 4844001..4845000 | 0.879733 | 1.840034734 |
| 4845001..4846000 | 0.818199 | 1.763203509 |
| 4846001..4847000 | 1.059978 | 2.084899728 |
| 4847001..4848000 | 0.987474 | 1.982710443 |
| 4848001..4849000 | 0.760966 | 1.694624932 |
| 4849001..4850000 | 1.037041 | 2.052014606 |
| 4850001..4851000 | 0.876138 | 1.835455325 |
| 4851001..4852000 | 1.045974 | 2.06475986 |
| 4852001..4853000 | 1.15895 | 2.232948535 |
| 4853001..4854000 | 1.3327 | 2.518736149 |
| 4854001..4855000 | 1.008914 | 2.012395683 |
| 4855001..4856000 | 0.965318 | 1.952493844 |
| 4856001..4857000 | 1.029548 | 2.04138458 |
| 4857001..4858000 | 0.877047 | 1.836612157 |
| 4858001..4859000 | 0.742259 | 1.672793081 |
| 4859001..4860000 | 0.83072 | 1.778572766 |
| 4860001..4861000 | 1.050613 | 2.071409801 |
| 4861001..4862000 | 0.796959 | 1.737434993 |
| 4862001..4863000 | 0.804937 | 1.747069499 |
| 4863001..4864000 | 1.024787 | 2.034658964 |
| 4864001..4865000 | 0.97885 | 1.970893745 |
| 4865001..4866000 | 0.886 | 1.848045145 |
| 4867001..4868000 | 0.881253 | 1.841974386 |
| 4868001..4869000 | 0.972301 | 1.961967305 |
| 4869001..4870000 | 0.814469 | 1.758650742 |
| 4870001..4871000 | 0.777158 | 1.713751585 |
| 4871001..4872000 | 0.79607 | 1.736364702 |
| 4872001..4873000 | 0.876734 | 1.836213738 |
| 4873001..4874000 | 0.997409 | 1.996411335 |
| 4874001..4875000 | 0.875026 | 1.834041139 |
| 4875001..4876000 | 0.933953 | 1.910503632 |
| 4876001..4877000 | 0.903157 | 1.870153902 |
| 4877001..4878000 | 0.785796 | 1.72404329 |
| 4878001..4879000 | 1.036746 | 2.051595056 |
| 4879001..4880000 | 0.926721 | 1.900950546 |
| 4880001..4881000 | 0.653716 | 1.573215167 |
| 4881001..4882000 | 0.676645 | 1.598418294 |
| 4882001..4883000 | 0.955699 | 1.939519122 |
| 4883001..4884000 | 1.031274 | 2.043828297 |
| 4884001..4885000 | 1.098279 | 2.140991399 |
| 4885001..4886000 | 0.777463 | 1.714113927 |
| 4886001..4887000 | 0.958236 | 1.942932795 |
| 4887001..4888000 | 1.152897 | 2.223599557 |
| 4888001..4889000 | 1.144369 | 2.210494278 |
| 4889001..4890000 | 0.899497 | 1.865415487 |
| 4890001..4891000 | 0.741095 | 1.671443977 |
| 4891001..4892000 | 0.719014 | 1.646056664 |
| 4892001..4893000 | 0.952396 | 1.93508374 |
| 4893001..4894000 | 0.971988 | 1.961541692 |
| 4894001..4895000 | 1.178954 | 2.264125612 |
| 4895001..4896000 | 1.182165 | 2.269170478 |
| 4896001..4897000 | 1.020933 | 2.029230852 |
| 4897001..4898000 | 0.977342 | 1.968834713 |
| 4898001..4899000 | 0.806556 | 1.74903117 |
| 4899001..4900000 | 0.665057 | 1.585630919 |
| 4900001..4901000 | 0.983709 | 1.977542911 |
| 4901001..4902000 | 0.842709 | 1.793414541 |
| 4902001..4903000 | 1.016246 | 2.022649023 |
| 4903001..4904000 | 0.835739 | 1.784771028 |
| 4904001..4905000 | 0.879489 | 1.839723559 |
| 4905001..4906000 | 1.17715 | 2.261296234 |
| 4906001..4907000 | 0.997755 | 1.996890189 |
| 4907001..4908000 | 1.161599 | 2.237052322 |
| 4908001..4909000 | 0.946132 | 1.926700064 |
| 4909001..4910000 | 0.868337 | 1.82555736 |
| 4912001..4913000 | 0.712024 | 1.638100646 |
| 4913001..4914000 | 0.702783 | 1.627641533 |
| 4914001..4915000 | 1.150497 | 2.219903556 |
| 4915001..4916000 | 1.012316 | 2.017146686 |
| 4916001..4917000 | 0.973277 | 1.963295048 |
| 4917001..4918000 | 0.81658 | 1.761225942 |
| 4918001..4919000 | 0.755851 | 1.688627366 |
| 4919001..4920000 | 1.121585 | 2.175858894 |
| 4920001..4921000 | 1.255406 | 2.387343232 |
| 4921001..4922000 | 0.852869 | 1.806089016 |
| 4922001..4923000 | 1.057714 | 2.081630492 |
| 4924001..4925000 | 0.896887 | 1.862043788 |
| 4925001..4926000 | 0.740779 | 1.671077913 |
| 4926001..4927000 | 0.877534 | 1.837232233 |
| 4927001..4928000 | 0.809314 | 1.752377991 |
| 4928001..4929000 | 0.769815 | 1.705051127 |
| 4929001..4930000 | 0.955857 | 1.939731545 |
| 4930001..4931000 | 0.989996 | 1.986179484 |
| 4931001..4932000 | 0.946995 | 1.927852934 |
| 4932001..4933000 | 1.113116 | 2.163123446 |
| 4933001..4934000 | 1.019026 | 2.026550322 |
| 4934001..4935000 | 0.849127 | 1.80141053 |
| 4935001..4936000 | 1.314201 | 2.48664576 |
| 4936001..4937000 | 0.948968 | 1.93049123 |
| 4937001..4938000 | 0.926481 | 1.900634339 |
| 4938001..4939000 | 0.777113 | 1.713698131 |
| 4939001..4940000 | 1.110767 | 2.159604308 |
| 4940001..4941000 | 0.654912 | 1.574519909 |
| 4941001..4942000 | 0.955753 | 1.93959172 |
| 4942001..4943000 | 0.926037 | 1.900049495 |
| 4943001..4944000 | 0.884633 | 1.846294892 |
| 4944001..4945000 | 0.937101 | 1.914676954 |
| 4945001..4946000 | 0.769945 | 1.705204775 |
| 4946001..4947000 | 1.004923 | 2.006836385 |
| 4947001..4948000 | 0.987545 | 1.982808021 |
| 4950001..4951000 | 0.644516 | 1.563214766 |
| 5295001..5296000 | -0.699161 | 0.615930297 |
| 5353001..5354000 | -0.704317 | 0.613732973 |
| 5935001..5936000 | -0.728174 | 0.603667484 |
| 6298001..6299000 | -0.881618 | 0.54275838 |
| 6312001..6313000 | -0.785118 | 0.580304491 |
