# Supplementary material for: Genomic Plasticity Enables Phenotypic Variation of Pseudomonas syringae pv. tomato DC3000
Source: PLoS One. 2014 Feb 6;9(2):e86628. doi: 10.1371/journal.pone.0086628 (PMC3916326; doi:10.1371/journal.pone.0086628)
Supplement: Table S3 — Genes and functional role categories contained within the 165 kb duplication. Multiple JCVI categories are separated by a “/”. In instances where a gene does not have a JCVI category assigned, the primary genbank annotation is shown. Genes annotated as having roles in pathogenesis are indicated with “♦”. Genes in the same functional role category are indicated by the color of background shading. *Disrupted reading frame. ** No JCVI Cellular role category information. (DOCX) [file pone.0086628.s009.docx]

| **Locus Tag** | **JCVI Cellular Role Category** | | | **Pathogenesis** |
| --- | --- | --- | --- | --- |
| PSPTO_4251 | Mobile and extrachromosomal element functions: Transposon functions | | |  |
| PSPTO_4252 | Mobile and extrachromosomal element functions: Transposon functions | | |  |
| PSPTO_4253 | Mobile and extrachromosomal element functions: Transposon functions | | |  |
| *gor-2* | Energy metabolism: Electron transport/Cellular processes: Pathogenesis | | | **◆** |
| PSPTO_4255 | Energy metabolism: Other | | |  |
| PSPTO_4256 | Unknown function: Enzymes of unknown specificity | | |  |
| PSPTO_4257 | Unknown function: Enzymes of unknown specificity | | |  |
| PSPTO_4258 | Energy metabolism: Electron transport | | |  |
| *kefC* | Transport and binding proteins: Cations and iron carrying compounds | | |  |
| *trxA* | Energy metabolism: Electron transport | | |  |
| PSPTO_4261 | Unknown function: Enzymes of unknown specificity | | |  |
| PSPTO_4262 | Regulatory functions: DNA interactions | | |  |
| PSPTO_4263 | Unknown function: Enzymes of unknown specificity | | |  |
| PSPTO_4264 | Hypothetical protein: Conserved | | |  |
| *metC-2* | Amino acid biosynthesis: Aspartate family | | |  |
| PSPTO_4266 | Cell envelope: Other | | |  |
| PSPTO_4267 | Regulatory functions: DNA interactions | | |  |
| PSPTO_4268* | Disrupted reading frame; Transport and binding proteins: Anions | | |  |
| PSPTO_4269 | Mobile and extrachromosomal element functions: Transposon functions | | |  |
| PSPTO_4270 | Mobile and extrachromosomal element functions: Transposon functions | | |  |
| PSPTO_4271* | Disrupted reading frame; Transport and binding proteins: Anions | | |  |
| PSPTO_4272 | Hypothetical protein | | |  |
| PSPTO_4273 | Disrupted reading frame; Mobile and extrachromosomal element functions: Prophage functions | | | |
| PSPTO_4274 | Regulatory functions: DNA interactions | | |  |
| PSPTO_4275 | Unknown function: Enzymes of unknown specificity | | |  |
| PSPTO_4276 | Regulatory functions: DNA interactions | | |  |
| PSPTO_4277 | Unknown function: Enzymes of unknown specificity | | |  |
| PSPTO_4278 | Transport and binding proteins: Unknown substrate | | |  |
| PSPTO_4279 | Hypothetical protein: Conserved | | |  |
| PSPTO_4280 | Cell envelope: Other | | |  |
| PSPTO_4281 | Hypothetical protein: Conserved | | |  |
| PSPTO_4282 | Hypothetical protein | | |  |
| *pnlA* | Cell envelope: Biosynthesis and degradation of surface polysaccharides and lipopolysaccharides/Cellular processes: Pathogenesis | | | ◆ |
| PSPTO_4284 | Unknown function: Enzymes of unknown specificity | | |  |
| *adhB* | Energy metabolism: Fermentation | | |  |
| PSPTO_4286 | Hypothetical protein: Conserved | | |  |
| PSPTO_4287 | Hypothetical protein | | |  |
| PSPTO_4288 | Hypothetical protein: Conserved | | |  |
| PSPTO_4289 | Hypothetical protein: Conserved | | |  |
| *bglX* | Energy metabolism: Biosynthesis and degradation of polysaccharides | | |  |
| PSPTO_4291 | Regulatory functions: Protein interactions/Signal transduction: Two-component systems | | |  |
| PSPTO_4292 | Regulatory functions: DNA interactions/Regulatory functions: Protein interactions/Signal transduction: Two-component systems | | | |
| PSPTO_4293 | Regulatory functions: Protein interactions/Signal transduction: Two-component systems | | |  |
| PSPTO_4294 | Protein fate: Protein folding and stabilization | | |  |
| PSPTO_4295 | Protein fate: Protein folding and stabilization | | |  |
| PSPTO_4296 | Transport and binding proteins: Unknown substrate | | |  |
| PSPTO_4297 | Hypothetical protein: Conserved domain | | |  |
| PSPTO_4298 | Hypothetical protein: Conserved | | |  |
| PSPTO_4299 | Hypothetical protein | | |  |
| PSPTO_4300 | Cellular processes: Toxin production and resistance/Transport and binding proteins: Other | | |  |
| PSPTO_4301 | Hypothetical protein: Conserved | | |  |
| PSPTO_4302 | Regulatory functions: DNA interactions | | |  |
| PSPTO_4303 | Transport and binding proteins: Unknown substrate | | |  |
| PSPTO_4304 | Cellular processes: Toxin production and resistance/Transport and binding proteins: Other | | |  |
| PSPTO_4305 | Transport and binding proteins: Unknown substrate | | |  |
| *pcaT* | Transport and binding proteins: Carbohydrates, organic alcohols, and acids/Energy metabolism: Other | | |  |
| *catF* | Energy metabolism: Other | | |  |
| *catJ* | Energy metabolism: Other | | |  |
| *catI* | Energy metabolism: Other | | |  |
| *pcaR* | Regulatory functions: DNA interactions | | |  |
| PSPTO_5644** | Hypothetical protein | | |  |
| PSPTO_4312 | Transport and binding protein: Anions | | |  |
| PSPTO_4313 | Hypothetical protein | | |  |
| *purU-3* | Purines, pyrimidines, nucleosides, and nucleotides: Purine ribonucleotide biosynthesis | | |  |
| PSPTO_4315 | Regulatory functions: DNA interactions | | |  |
| *sbcB* | DNA metabolism: Degradation of DNA/DNA metabolism: DNA replication, recombination, and repair | | | |
| PSPTO_4317 | Hypothetical protein: Conserved | | |  |
| PSPTO_4318 | Cell envelope: Other | | |  |
| PSPTO_4319 | Hypothetical protein: Conserved | | |  |
| PSPTO_4320 | Hypothetical protein: Conserved | | |  |
| PSPTO_4321 | Hypothetical protein | | |  |
| PSPTO_4322 | Hypothetical protein | | |  |
| PSPTO_4323 | Hypothetical protein: Conserved | | |  |
| PSPTO_4324 | Hypothetical protein | | |  |
| PSPTO_4325 | Hypothetical protein | | |  |
| PSPTO_4326 | Hypothetical protein: Conserved | | |  |
| PSPTO_4327 | Hypothetical protein | | |  |
| PSPTO_4328 | Mobile and extrachromosomal element functions: Transposon functions | | |  |
| PSPTO_4329 | Mobile and extrachromosomal element functions: Transposon functions | | |  |
| PSPTO_4330 | Hypothetical protein: Conserved domain | | |  |
| *hopE1* | Protein fate: Protein and peptide secretion and trafficking/Cellular processes: Pathogenesis | | | ◆ |
| PSPTO_4332 | Hypothetical protein: Conserved | | |  |
| PSPTO_4333 | Unknown function: General | | |  |
| PSPTO_4334 | Hypothetical protein: Conserved | | |  |
| PSPTO_4335 | Hypothetical protein: Conserved | | |  |
| PSPTO_4336 | Cell envelope: Other | | |  |
| *pyk* | Energy metabolism: Glycolysis/gluconeogenesis | | | ⬝ |
| PSPTO_4338 | Unknown function: Enzymes of unknown specificity | | |  |
| PSPTO_4339 | Energy metabolism: TCA cycle | | |  |
| PSPTO_4340 | Cellular processes: Toxin production and resistance | | |  |
| PSPTO_4341 | Cellular processes: Toxin production and resistance | | |  |
| PSPTO_4342 | Cellular processes: Toxin production and resistance | | |  |
| PSPTO_4343 | Cellular processes: Toxin production and resistance | | |  |
| PSPTO_4344 | Cellular processes: Toxin production and resistance | | |  |
| PSPTO_4345 | Hypothetical protein: Conserved | | |  |
| PSPTO_4346 | Hypothetical protein | | |  |
| PSPTO_4347 | Mobile and extrachromosomal element functions: Transposon functions | | |  |
| PSPTO_4348 | Hypothetical protein | | |  |
| PSPTO_4349 | Unknown function: General | | |  |
| PSPTO_4350 | Unknown function: General | | |  |
| PSPTO_4351 | Hypothetical protein: Conserved | | |  |
| PSPTO_4352 | Unknown function: Enzymes of unknown specificity | | |  |
| PSPTO_4353 | Hypothetical protein: Conserved | | |  |
| PSPTO_4354 | Unknown function: General | | |  |
| PSPTO_4355 | Hypothetical proteins: Conserved | | |  |
| PSPTO_4356 | Regulatory functions: DNA interactions | | |  |
| PSPTO_4357 | Transport and binding proteins: Unknown substrate | | |  |
| PSPTO_4358 | Unknown function: Enzymes of unknown specificity | | |  |
| PSPTO_4359 | Hypothetical protein: Domain | | |  |
| PSPTO_4360 | Unknown function: Enzymes of unknown specificity | | |  |
| *iciA* | DNA metabolism: DNA replication, recombination, and repair/Regulatory functions: DNA interactions | | | |
| PSPTO_4362 | Transport and binding proteins: Amino acids, peptides and amines | | |  |
| *sodB* | Cellular processes: Detoxification/Cellular processes: Pathogenesis | | | ◆ |
| PSPTO_4364 | Hypothetical protein | | |  |
| PSPTO_4365 | Unknown function: General | | |  |
| PSPTO_4366 | Cell envelope: Other | | |  |
| PSPTO_4367 | Cell envelope: Other | | |  |
| PSPTO_4368 | Cell envelope: Other | | |  |
| PSPTO_4369 | Cell envelope: Other | | |  |
| PSPTO_4370 | Hypothetical protein: Conserved | | |  |
| *inaA* | Regulatory functions: Other | | |  |
| PSPTO_4372 | Hypothetical protein | | |  |
| *colS* | Regulatory functions: Protein interactions/Signal transduction: Two-component systems | | |  |
| *colR* | Regulatory functions: DNA interactions/Regulatory functions: Protein interactions/Signal transduction: Two-component systems | | | |
| PSPTO_4375 | Unknown function: General | | |  |
| *groEL* | Protein fate: Protein folding and stabilization | | |  |
| *groES* | Protein fate: Protein folding and stabilization | | |  |
| PSPTO_4378 | Hypothetical protein | | |  |
| PSPTO_4379 | Unknown function: General | | |  |
| PSPTO_4380 | Unknown function: Enzymes of unknown specificity | | |  |
| PSPTO_4381 | Hypothetical protein: Conserved | | |  |
| PSPTO_4382 | Unknown function: General | | |  |
| PSPTO_4383 | Transport and binding proteins: Unknown substrate | | |  |
| PSPTO_4384 | Hypothetical protein | | |  |
| PSPTO_4385 | Unknown function: General | | |  |
| PSPTO_4386 | Hypothetical protein | | |  |
| PSPTO_4387 | Hypothetical protein | | |  |
| PSPTO_4388 | Hypothetical protein | | |  |
| PSPTO_4389 | Mobile and extrachromosomal element functions: Transposon functions | | |  |
| PSPTO_4390 | Mobile and extrachromosomal element functions: Transposon functions | | |  |
| Legend | |  |  |  |
| Functional role categories | | No. of genes per category |  |  |
| Mobile and extrachromosomal element functions/ Prophage functions | | 11 |  |  |
| Energy metabolism | | 11 |  |  |
| Unknown function/Hypothetical proteins | | 66 |  |  |
| Transport and binding proteins/Cell envelope | | 21 |  |  |
| Regulatory functions: DNA interactions/Two component Regulators | | 14 |  |  |
| Amino acid biosynthesis | | 1 |  |  |
| Protein fate: Protein folding and stabilization | | 5 |  |  |
| Nucleic acid metabolism | | 3 |  |  |
| Toxin production and resistance | | 8 |  |  |
| *Disrupted reading frame | | Total = 140 |  |  |
| ** No JCVI Cellular role category information | |  |  |  |
